# Whole genome sequencing and characterization of *Pantoea agglomerans* DBM 3797, endophyte, isolated from fresh hop (*Humulus lupulus* L.)

**DOI:** 10.3389/fmicb.2024.1305338

**Published:** 2024-02-08

**Authors:** Petra Patakova, Maryna Vasylkivska, Karel Sedlar, Katerina Jureckova, Matej Bezdicek, Petra Lovecka, Barbora Branska, Petr Kastanek, Karel Krofta

**Affiliations:** ^1^Department of Biotechnology, University of Chemistry and Technology Prague, Prague, Czechia; ^2^Department of Biomedical Engineering, Faculty of Electrical Engineering and Communication, Brno University of Technology, Brno, Czechia; ^3^Department of Informatics, Ludwig-Maximilians-Universität München, Munich, Germany; ^4^Department of Internal Medicine—Hematology and Oncology, University Hospital Brno, Brno, Czechia; ^5^Department of Internal Medicine–Hematology and Oncology, Faculty of Medicine, Masaryk University, Brno, Czechia; ^6^Department of Biochemistry and Microbiology, University of Chemistry and Technology Prague, Prague, Czechia; ^7^Ecofuel Laboratories s.r.o., Prague, Czechia; ^8^Hop Research Institute, Co. Ltd., Zatec, Czechia

**Keywords:** *Pantoea agglomerans*, hops endophyte, plant growth promotion, genome characterization, gluconic acid

## Abstract

**Background:**

This paper brings new information about the genome and phenotypic characteristics of *Pantoea agglomerans* strain DBM 3797, isolated from fresh Czech hop (*Humulus lupulus*) in the Saaz hop-growing region. Although *P. agglomerans* strains are frequently isolated from different materials, there are not usually thoroughly characterized even if they have versatile metabolism and those isolated from plants may have a considerable potential for application in agriculture as a support culture for plant growth.

**Methods:**

*P. agglomerans* DBM 3797 was cultured under aerobic and anaerobic conditions, its metabolites were analyzed by HPLC and it was tested for plant growth promotion abilities, such as phosphate solubilization, siderophore and indol-3-acetic acid productions. In addition, genomic DNA was extracted, sequenced and *de novo* assembly was performed. Further, genome annotation, pan-genome analysis and selected genome analyses, such as CRISPR arrays detection, antibiotic resistance and secondary metabolite genes identification were carried out.

**Results and discussion:**

The typical appearance characteristics of the strain include the formation of symplasmata in submerged liquid culture and the formation of pale yellow colonies on agar. The genetic information of the strain (in total 4.8 Mb) is divided between a chromosome and two plasmids. The strain lacks any CRISPR-Cas system but is equipped with four restriction-modification systems. The phenotypic analysis focused on growth under both aerobic and anaerobic conditions, as well as traits associated with plant growth promotion. At both levels (genomic and phenotypic), the production of siderophores, indoleacetic acid-derived growth promoters, gluconic acid, and enzyme activities related to the degradation of complex organic compounds were found. Extracellular gluconic acid production under aerobic conditions (up to 8 g/l) is probably the result of glucose oxidation by the membrane-bound pyrroloquinoline quinone-dependent enzyme glucose dehydrogenase. The strain has a number of properties potentially beneficial to the hop plant and its closest relatives include the strains also isolated from the aerial parts of plants, yet its safety profile needs to be addressed in follow-up research.

## 1 Introduction

The Czech Republic is famous for its Pilsner beer, in which hops (*Humulus lupulus* L.) is irreplaceable feedstock. Hops (*Humulus lupulus* L.) is a perennial, dioecious, climbing plant belonging to the *Cannabaceae* family and order of *Rosales* (Zhang et al., 2011). Hop cones of the female plant contain in lupulin glands a lot of secondary metabolites which are mostly used in beer production. Hop resins, essential oils and their transformation products impart beer its typical bitter taste and hoppy aroma (Jaskula et al., 2008). A number of substances contained in hops have at the same time many biologically active effects. 8-Prenylnaringenin is known to be the most potent phytoestrogen to date (Milligan et al., 2000). Beta acids are characterized by strong antimicrobial effects against some groups of bacteria (Sleha et al., 2021; Fahle et al., 2022). Xanthohumol from the group of prenylated flavonoids has anticarcinogenic effects against certain types of cancer (Miranda et al., 1999).

Till now, hop plant research has focused on topics other than the natural colonization of the hop plant by endo- and epiphytic bacteria, with only a few exceptions (Goryluk-Salmonovicz et al., 2016; Allen et al., 2019; Micci et al., 2022). On the contrary, it was rather assumed that hop would not be colonized by bacteria because many of its metabolites have antimicrobial properties (for a review see, Bocquet et al., 2018). The considerable resistance of hops to bacteria is also evidenced by the fact that bacterial diseases of hops are rare compared to viruses or diseases caused by fungi. Nevertheless, some bacteria have already been isolated from hops—bacteria of the genus *Streptomyces* from the rhizosphere of hops (Koçak, 2019), *Pseudomonas stutzeri* and *Pseudomonas fluorescens* from hop cones (Sevigny et al., 2019) and *Pantoea agglomerans* from hop cones (Sevigny et al., 2019) and from dried hop pellets (Kolek et al., 2021).

Various *Pantoea* species and strains have been isolated as free-living bacteria from different habitats or from different hosts, having loose or tighter relationships to the host, i.e., many of them being plant epiphytes/endophytes, sometimes plant pathogens, others being insect symbionts or facultative human pathogens (Walterson and Stavrinides, 2015). Specifically, different strains of *P. agglomerans* were isolated from different plants (Walterson and Stavrinides, 2015) or were found as clinical isolates causing various health problems (Soutar and Stavrinides, 2019). In the same time, other *P. agglomerans* strains had beneficial properties in medicine (such as macrophage activation or to combat *Plasmodium* parasites) while yet other strains can become biocontrol agents or mediate improved plant nutrition, which might be very useful for future sustainable agricultural practice (Dutkiewicz et al., 2016). To identify differences between plant and clinical isolates through their genomes is difficult (Rezzonico et al., 2009) and is complicated by changing taxonomy and frequent misidentification of *P. agglomerans* clinical isolates (Rezzonico et al., 2009; Soutar and Stavrinides, 2019). Regarding taxonomy, into the species name *P. agglomerans* were transferred all older species originally called *Enterobacter agglomerans* or *Erwinia herbicola* and these species names are used as synonyms now. However, older isolates of *E. agglomerans* or *E. herbicola* frequently differ from *P. agglomerans* (Soutar and Stavrinides, 2019).

Despite a number of published materials, little attention has been devoted to the fact that *P. agglomerans*, as a facultative anaerobic bacterium, behaves very differently under different conditions and can switch between different metabolic pathways. In particular its anaerobic metabolism has been neglected, but can harbor surprises. *P. agglomerans* DBM 3696 was identified as the probable causative agent of inflation (production of CO_2_) in bags filled with dried hop pellets stored in a modified atmosphere (Kolek et al., 2021). This study aims to demonstrate the versatile metabolism of *P. agglomerans* DBM 3697 isolated from fresh green hop cones in the Steknik hopyards (Czech Republic) along with identification of significant metabolites, as well as the complete genome and its comprehensive analysis, stressing potentially beneficial properties that may be used in agriculture (e.g., phosphate solubilisation, siderophore and auxin (indol-3-acetic acid and its derivatives) productions and others).

Currently, about 139 *P. agglomerans* genome reports can be found in the NCBI GenBank/RefSeq database, but in only 31 cases, complete genome sequences have been published.^1^ Regarding plant associated *P. agglomerans*, not showing pathogenesis, comprehensive genome analyses were performed for only five strains shown in Table 1. The complete available genomic dataset of different *P. agglomerans* strains, although it may seem extensive at first glance, is in fact insufficient for differential analysis of the genomes to find significant differences between beneficial and pathogenic strains. To be able to do so, it is necessary to expand this dataset to include strains that do not show pathogenesis to plants or humans, as well as clinical isolates or strains associated with plant pathogenesis.

**Table 1:** Comprehensive genome analyses of plant associated non-pathogenic *P. agglomerans*.

**Strain**	**Plant association**	**Genome information**	**Specific features**	**Reference**
P5	Soil sample	5.04 Mb Scaffold assembly	Potential biofertilizer	Shariati et al., 2017
C1	Isolated from the phyllosphere of lettuce (*Lactuca sativa*)	4.85 Mb 21 contigs	Plant growth-promoting (PGP) bacterium in heavy metal polluted soils	Luziatelli et al., 2020a
ANP8	Isolated from root nodules of alfalfa (*Medicago sativa*) grown in saline soil	5.03 Mb Scaffold assembly	PGP activities in saline soil	Noori et al., 2021
CPHN2	Isolated from chickpea (*Cicer arietinum*) non-rhizobial nodule	4.8 Mb (chromosome and 2 plasmids) 32 contigs	Potential biofertilizer	Kumar et al., 2022
DAPP-PG 734	Endophytic bacterium, isolated from knots (tumors) of olive tree (*Olea europaea*)	5.4 Mb (chromosome and 4 plasmids) Five contigs	Potential biocontrol activity	Sulja et al., 2022
DBM 3797	Isolated from fresh green hop (*Humulus lupulus*) cones	4.8 Mb (chromosome and two plasmids) Complete genome	PGP activities	This study

## 2 Material and methods

### 2.1 The strain isolation and its storage

The strain *Pantoea agglomerans* DBM 3797, deposited in the culture collection of the Department of Biochemistry and Microbiology (DBM) of the UCT Prague, was stored at −80°C. The strain was isolated from fresh hop cones grown in Steknik hopyards in the Czech Republic (altitude 192 m,_latitude and longitude: 50.3166292 N 13.6102039 E). The plant material was collected under aseptic conditions in a sterile plastic bag, surface sterilization with ethanol was performed in the laboratory, the material was ground and suspended in sterile physiological solution (0.9% NaCl). The solid particles were then filtered under aseptic conditions through sterile folded filter paper and the filtrate was diluted 10 ×, 100 × and 1,000 ×. From each dilution, 0.1 ml was inoculated onto the surface of the solidified LB medium. The plates were incubated for 24 or 48 h at 30°C. From the initial growth, the culture was plated several times on the surface of the agar medium and individual colonies were isolated.

### 2.2 Culture conditions

All chemicals for preparation of culture media, as well as for microbiological assays of plant growth promoting activities, were purchased from Merck if not stated otherwise. The strain was cultured in Lysogeny Broth (LB) culture medium containing (g/l): tryptone 10, yeast extract 5 and NaCl 5 or in Pantoea glucose medium (PGM) containing (g/l): glucose 10 or 20, MgSO_4_.7H_2_O 0.4, NaCl 1, CaCl_2_.2H_2_O 0.2, NH_4_NO_3_ 1.5; yeast extract 0.2, KCl 0.2, peptone 0.5. The ability to utilize different carbon sources was tested in PGM, where glucose was changed for xylose, cellulose (Avicel) or lignin, always at a concentration of 10 g/l. For bioreactor culture, the glucose concentration was 20 g/l. In some experiments, LB culture medium was supplemented with glucose at a concentration of 10 g/l. For growth in Petri dishes, culture medium was supplemented with 20 g/l of agar. For indole-3-acetic acid production, tryptophan was added to the culture medium at a concentration of 5 g/l. Culture experiments were performed at 30°C for 24–48 h. Each inoculum for culture experiments was prepared by overnight growth in LB liquid medium.

Cultivation experiments were run in Erlenmeyer shake flasks on a rotary shaker (150 rpm), in a 1 l bioreactor (Infors HT), both aerobically and anaerobically, in a thermostat (the case of growth on solidified medium in Petri dishes) or in an anaerobic chamber (Concept 400, UK). For bioreactor experiments, PGM culture medium with 20 g/l glucose was used. The working volume of the 1 l bioreactor was 700 ml (630 ml of fresh culture medium and 70 ml of inoculum) and pH monitoring were used. In aerobic culture, the filtered air rate was 1 VVM and oxygen saturation was measured using an oxygen electrode. Details of anaerobic bioreactor culture were described previously by Sedlar et al. (2021).

### 2.3 Analyses

Growth was monitored as an optical density (OD) at 600 nm using a spectrophotometer (Agilent Cary 60 UV-VIS) against the respective medium without inoculation as a blank. Microscopic control of the culture was performed using phase contrast microscopy (Olympus BX51; Olympus).

The concentration of substrate (glucose) and metabolites (ethanol, lactic, acetic and gluconic acids) were determined by HPLC (Agilent Series 1200 HPLC; Agilent) with refractive index detection. The parameters of the HPLC analysis were as follows: injection sample volume of 20 μl, 5 mM H_2_SO_4_ as a mobile phase, a flow rate of 1 ml/min, IEX H^+^ polymer column (Watrex) and a column temperature of 60°C.

Statistical analysis of different growth conditions was performed in R (v4.3.1). Data normality was checked with Shapiro-Wilk test (*p*-value < 0.05) and homogeneity of variance was verified using Bartlett's test (*p*-value < 0.05). One-way ANOVA with *post-hoc* Tukey test was performed at *p*-adjusted value < 0.05 to identify statistically significant changes under different cultivation conditions.

### 2.4 DNA extraction and sequencing

For short-read sequencing, genomic DNA was extracted and purified using the GenElute Bacterial Genomic DNA Kit (Sigma-Aldrich, St. Louis, MI, USA) following the manufacturer's protocols. The purity of the DNA was assessed using a NanoDrop spectrophotometer (Thermo Scientific, Wilmington, DE, USA), while the concentration was determined using the Qubit 3.0 (Thermo Scientific, Wilmington, DE, USA). DNA library construction was carried out using the KAPA HyperPlus kit, following the standard protocol. Subsequently, sequencing was performed on the Illumina MiSeq platform (Illumina, San Diego, CA, USA) using the MiSeq Reagent Kit v2 (500 cycles).

For long-read sequencing, high molecular weight genomic DNA was extracted using the MagAttract HMW DNAKit (Qiagene, Venlo. NL). The purity of the extracted DNA was assessed with the NanoDrop (Thermo Fisher Scientific, Waltham, MA, USA), while the concentration was determined using the Qubit 3.0 (Thermo Scientific, Wilmington, DE, USA). The DNA length was confirmed using the Agilent 4200 TapeStation (Agilent Technologies, Santa Clara, CA, USA). Ligation sequencing 1D Kit (Oxford Nanopore Technologies, Oxford, UK) was used for library preparation, and sequenced on the MinION platform (Oxford Nanopore Technologies) with the R9.4.1 flowcell.

### 2.5 Genome assembly

Long Oxford Nanopore Technologies (ONT) reads were basecalled with Guppy v3.4.4 and used for the initial *de novo* assembly performed with Flye v2.8.1. The assembly was polished with Racon v1.4.13 (Vaser et al., 2017) and Medaka v1.2.5 using ONT reads quality checked with MinIONQC (Lanfear et al., 2019). Auxiliary PAF files were generated using minimap2 (Li, 2018). Short Illumina paired reads were quality trimmed with Trimomatic v1.36 (Bolger et al., 2014), checked with FastQC v0.11.5 and MultiQC v1.7 (Ewels et al., 2016), and used for additional rounds of polishing with Pilon v1.24 (Walker et al., 2014). For that purpose, short reads were mapped onto ONT assembly with BWA v07.17 (Li and Durbin, 2009) and auxiliary BAM files were processed with SAMtools (Li et al., 2009). Finally, the resulting chromosomal sequence was rearranged according to the origin of replication (*oriC*) to *dnaA*, being the first gene on the sense strand, using the Ori-finder (Luo et al., 2019) and both plasmid sequences were rearranged in a similar manner to the *repB* gene coding for plasmid replication initiator, being the first gene on the sense strand, using manual BLAST searches (Altschul et al., 1990).

### 2.6 Genome annotation and analysis

Genome annotation was performed by NCBI Prokaryotic Genome Annotation Pipeline (PGAP) (Tatusova et al., 2016). The functional annotation of protein coding genes was extended by classification into categories of clusters of orthologous groups (COG). Overall three sources of COG categories were used, namely eggNOG-mapper (Cantalapiedra et al., 2021) (v2.1.9), Operon-mapper (Taboada et al., 2018) and Batch CD-Search (Marchler-Bauer and Bryant, 2004) tools. Results were further processed by COGtools (v1.0.0) (https://github.com/xpolak37/COGtools) to merge them and create a final improved COG annotation. Assigned COG categories were visualized as circular plots by DNAplotter (Carver et al., 2009), which is a part of the Artemis (Carver et al., 2012) (v2.18.0) software. Selected pathogenic and non-pathogenic chromosomal sequences were compared and visualized as a circular graph in BRIG (v0.95) software (Alikhan et al., 2011). Pan-genome analysis was performed using BPGA v1.3 (Chaudhari et al., 2016), with amino acid sequences clustered using USEARCH (Edgar, 2010), with an identity cut-off of 90%. In total, 139 genomes of *P. agglomerans* were obtained from the NCBI RefSeq database (30th October 2023) (O'Leary et al., 2016) to define the core genome and to perform a phylogenomic analysis, i.e., concatenated sequences of core genes were aligned with MUSCLE and resulting multiple sequence alignment was used to reconstruct phylogeny with Neighbor-Joining algorithm using Kimura distance implemented in BPGA.

The genome was searched for clustered regularly interspaced short palindromic repeat (CRISPR) arrays using the CRISPRDetect (Biswas et al., 2016) (v2.4) tool and *cas* genes were searched in the genome manually. Components of restriction-modification (RM) systems were identified using REBASE (v307) database (Roberts et al., 2023). Prophage DNA was searched with the online version of PHASTER (Arndt et al., 2016). Antibiotic-resistant genes search was performed using Resistance Gene Identifier (RGI) 6.0.0 included in the Comprehensive Antibiotic Resistance Database (CARD) 3.2.5 (Alcock et al., 2020) by submitting protein sequences of CDSs. Virulence factors were searched using online version of VFAnalyzer against the virulence factor database (VFDB) (Liu et al., 2019) with default parameters and using *Klebsiella pneumoniae* as the closest annotated reference for *P. aglomerans*. Homologs of genes involved in biosynthetic pathways, putatively contributing to plant growth promotion and other activities were identified with tBLASTn, with the use of target protein sequences from closely related species. The length of initial seeds was set to 5 and BLOSUM62 matrix was used for scoring the alignments while gap introduction and extension was set to 11 and 1, respectively. Finally, identification of secondary metabolite biosynthesis gene clusters was performed with antiSMASH v7.1.0 (Blin et al., 2023) through its web service using relaxed detection strictness parameter.

### 2.7 Plant growth promoting activities

Screening of PGP activities was performed by established microbiological assays combined with spectrophotometric or visual detection and frequently (if not stated otherwise) at a semi-quantitative level (low, medium, or high).

Siderophore production was tested on blue agar chrome azurol S medium containing chrome azurol S and hexadecyltrimethylammonium bromide as indicators. Development of a yellowish orange halo around the colonies was taken as indicative of siderophore production; for details see Schmidt et al. (2018).

Phosphate solubilization was detected as a clear zone, i.e., the ability to solubilize calcium phosphate using Pikovskaya medium see Schmidt et al. (2018) or was tested in liquid NBRIP medium where the concentration of phosphate was determined spectrophotometrically by the ammonium molybdate-ascorbic acid method (Stranska et al., 2021).

Nitrogen fixation ability was tested in NFGM medium and evaluated spectrophotometrically; details are presented in Stranska et al. (2021).

Amylase, lipase, pectinase, protease/peptidase, and cellulase production were tested for in the appropriate solidified culture medium (Hawar, 2022) and evaluated as a halo or colored zone around a colony.

Ammonium release was detected after 24 h growth in LB medium by the Quantofix rapid test following instructions of the producer (Quantofix).

Indole-3-acetic acid (IAA) or IAA-like compound production was tested by Salkowski reagent (0.01 M FeCl_3_ in 35% HClO_4_) in LB culture medium supplemented with tryptophan after 48 h growth on a rotary shaker; for details of the procedure see Gilbert et al. (2018).

Indole production/release was tested by reaction with Kovacs reagent (Merck) in LB culture medium after 24 h growth on a rotary shaker.

## 3 Results

### 3.1 Genome and pan-genome

The genome of *P. agglomerans* DBM 3797 comprises a circular chromosome (size 4,089 kb) and two circular plasmids (pPA_DBM3797_1 size 555 kb and pPA_DBM3797_2 size 182 kb) assembled using both long reads and short reads in a hybrid approach with an overall coverage of 584 × and deposited at the DDBJ/EMBL/GenBank under accession numbers CP086133.1, CP086134.1, and CP086135.1, respectively. The overall genome length is 4,827,556 bp and contains 4,486 open reading frames (ORFs). While 4,328 ORFs present protein-coding sequences (CDSs), 49 genes had corrupted ORFs and formed pseudogenes. The remaining loci corresponded to RNA coding genes. Statistics for chromosome and both plasmids are summarized in Table 2. While most genes putatively corresponding to phenotypic traits were found on the chromosome, some of them were located on the large pPA_DBM3797_1 plasmid.

**Table 2:** Genome features of *P. agglomerans* DBM 3797.

**Feature**	**Chromosome**	**pPA_DBM3797_1**	**pPA_DBM3797_2**
Length (bp)	4 089 578	555 522	182 426
GC content (%)	55.5	52.5	53.0
ORFs	3,808	529	149
CDSs	3,673	508	147
Pseudogenes	26	21	2
rRNA genes (5S, 16S, 23S)	8, 7, 7	0, 0, 0	0, 0, 0
tRNAs	77	0	0
ncRNAs	10	0	0

**Figure 1:**
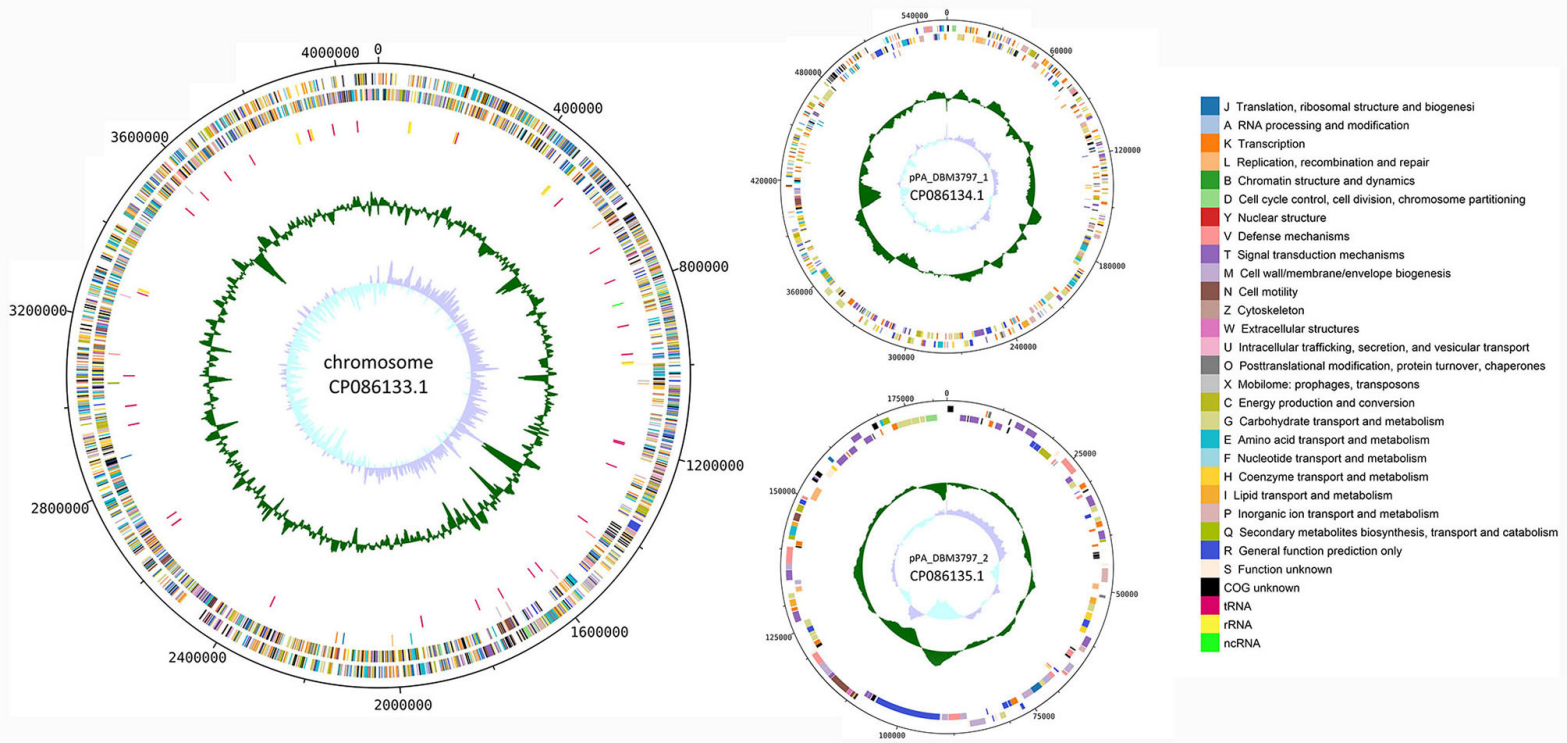
A circular map of the chromosome **(on the left)** and both plasmid **(on the right)** sequences of *P. agglomerans* DBM 3797. From outside to center: CDS on the forward strand (color-coded by COG categories), CDS on the reverse strand (color-coded by COG categories), pseudogenes (color-coded by COG categories), RNA genes (tRNA, rRNA, ncRNA), GC content and GC skew.

Functional annotation of the genome was done by classifying protein coding genes and pseudogenes into 26 categories of clusters of orthologous genes (COG), see Figure 1. For the chromosomal sequence, 3,280 genes were assigned a COG while 419 genes (11.33%) remained unannotated. Three most abundant categories were categories: E (Amino acid transport and metabolism) with 323 genes (8.73%), G (Carbohydrate transport and metabolism) with 321 genes (8.68%), and M (Cell wall/membrane/envelope biogenesis) with 268 genes (7.25%). For plasmid pPA_DBM3797_1 sequence, 66 genes (12.48%) remained unannotated and from 463 remaining genes, 78 genes (14.74%) were assigned category K (Transcription) and 69 genes (13.04%) category G (Carbohydrate transport and metabolism), forming the two most abundant groups. Moreover, 130 genes found on plasmid pPA_DBM3797_2 were assigned a COG while the most frequent was category T (Signal transduction mechanism) with 20 genes (13.42 %). Other abundant groups were formed by genes assigned to K (Transcription) and V (Defense mechanisms), both containing 15 genes (10.07%). Nineteen genes (12.75%) remained unannotated. For a complete summary of COG statistics see Supplementary Table 1. Additionally, seven secondary metabolite biosynthetic gene clusters were found. While five regions corresponding to redox-cofactor (2,192,097–2,214,263), arylpolyene and homoserine lactone (hserlactone) (2,614,136–2,673,970), thiopeptide (2,739,933–2,766,189), hserlactone (3,555,694–3,576,332), and NRP (non-ribosomal peptide)-metallophore and NRPS (non-ribosomal peptide synthase) (3,660,174–3,713,865) were located on the chromosome, remaining two regions corresponding to NI-siderophore (165,106–195,460) and terpene (376,789–400,350) were located on the pPA_DBM3797_1 plasmid (Supplementary Figure 1).

The *P. agglomerans* genome was missing any clustered regularly interspaced short palindromic repeat (CRISPR) arrays and similarly, no *cas* genes are present. Furthermore, we found four restriction-modification (R-M) systems, one was of type I and the remaining three were of type II. A type I R-M system consisted of one restriction enzyme Pag3797ORF16840P, two methyltransferases M1.Pag3797ORF16840P and M2.Pag3797ORF16840P, and one specificity subunit S.Pag3797ORF16840P. In all three type II systems, we found methyltransferases: M.Pag3797DamP, M.Pag3797ORF3130P and M.Pag3797DcmP, while the last enzyme was also coupled with nicking enzyme V.Pag3797DcmP. Complete results for R-M systems can be found in Supplementary Table 2. Only a single intact prophage of length 41.8 kbp corresponding to phage PHAGE_Erwini_ENT90_NC_019932 was found on the chromosome, within region 648492-690301. The whole region contained 57 proteins in total while 53 of these genes corresponded to phage DNA.

Last but not least, the genome was searched for antibiotic resistance and virulence genes. In total, 12 strict hits were found in the Comprehensive Antibiotic Resistance Database. While 11 genes were localized on chromosome, the remaining gene was found on plasmid pPA_DBM3797_2, see Supplementary Table 3. Six of these genes corresponded to antibiotic efflux resistance mechanisms, five were predicted to be responsible for antibiotic target alternation and one for antibiotic inactivation. The presence of these genes was confirmed by searching for virulence factors in general using the Virulence Factor Database. The presence of other virulence factors remained inconclusive as only partial hits to other secretion system or endotoxin genes were detected. The only complete system corresponded to gene machinery responsible for flagella construction, however, the cell motility is not necessarily connected to virulence.

The chromosomal sequence of *P. agglomerans* DBM 3797 was compared with chromosomal sequences of selected pathogenic and non-pathogenic strains downloaded from GenBank database under further mentioned accession numbers. These included the only pathogenic available strain isolated from clinical, FDAARGOS 1447 (CP077366.1); a plant pathogenic strain, BH6c (CP134744.1); and three non-pathogenic strains isolated from the same plant part (above-ground part) as DBM 3797, namely DAPP-PG734 (OW970315.1), CPHN 2(CP098414.1), and CFSAN047154 (CP034474.1). The results of a comparative analysis revealed no significant differences among the chromosomal sequences. All analyzed sequences were aligned to the reference strain DBM 3797 with 100% identity along almost the entire length of the sequence (see Supplementary Figure 2).

**Figure 2:**
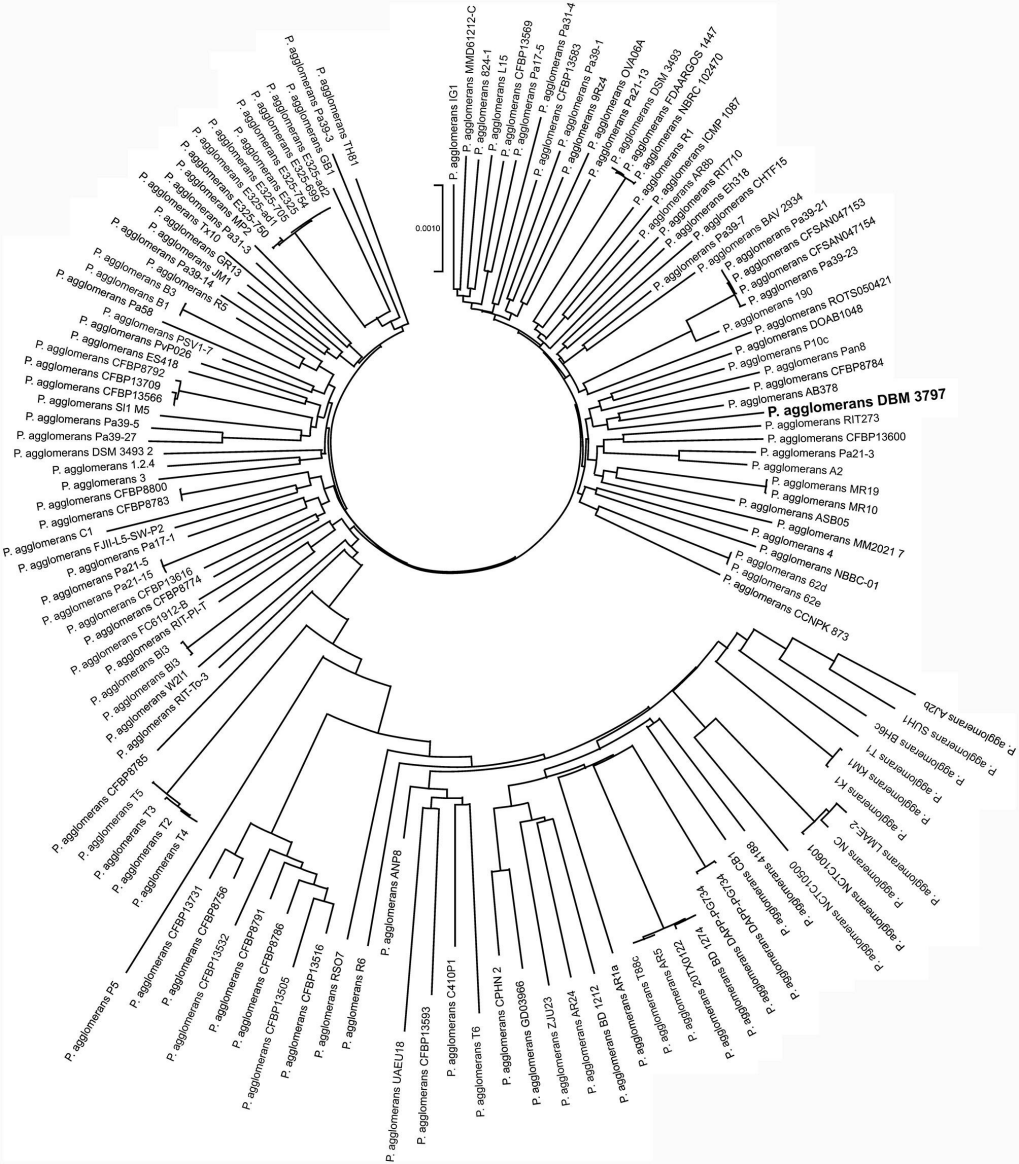
Phylogenomic analysis based on concatenated sequences of 2,399 core genes of 139 genomes of *P. agglomerans* strains. The tree was reconstructed using the Neighbor-Joining method using the Bacterial Pan Genome Analysis tool (BPGA).

The pan-genome analysis showed that all currently available genomes of *P. agglomerans* strains with successful taxonomy check shared 2,399 genes that formed the core genome of the species. Phylogenomic tree reconstructed using concatenated sequences of all core genes showed that *P. agglomerans* DBM 3797 presented a well-distinguished strain with strains AB378 and CFBP8784 being the closest relatives, see Figure 2. The complete list of the strains included into the Figure 2 is shown in Supplementary Table 4.

### 3.2 Growth, metabolite formation, and putative corresponding genes

*P. agglomerans* DBM 3797 was grown under aerobic and anaerobic conditions in liquid medium. Ability to grow in the presence/absence and limitation of oxygen requires security of basic life functions under both conditions, such as ability to synthesize deoxyribonucleotides by ribonucleotide reductases (RNR). RNRs mediate the reduction of nucleotides differently under aerobic/anaerobic conditions and for this, different enzymes are required. The corresponding RNR genes located on the chromosome are shown in Table 3. Under aerobic conditions, the strain preferred LB medium, which did not contain saccharides. Young cells (5 h after inoculation) were highly motile while in older LB-medium, in the grown population (after 24 h), symplasmata formation was observed (Supplementary Figure 3). Putative genes responsible for motility and symplasmata formation (biofilm like structure) were found on the chromosome and are shown in Supplementary Table 5. It was also tested whether symplasmata formation might be initiated by indole release during tryptophan degradation (tryptophan presence was assumed in LB medium) by the reaction of culture medium supernatant with Kovacs reagent, but the reaction was negative. In addition, the gene for tryptophanase was not found on the chromosome, nor on the plasmids. An interesting feature of growth in LB medium was alkalization of the culture medium (up to pH 8.7, see Table 4), which was caused by the release of ammonium ions from amino acids, serving as a carbon source in the medium. Ammonium ion concentration was about 100–200 mg/l. Aerobically, the culture was able to utilize glucose, xylose, cellulose (Avicel) and lignin, however compared to LB medium growth, the cells were shorter, the amount of biomass formed in 24 h was about 10 times lower and no symplasmata were observed. Anaerobic growth required saccharides for a fermentative way of obtaining energy and therefore was not possible in simple LB medium not containing glucose. On solidified LB medium under aerobic conditions, the strain formed round, convex, slimy looking, cream to yellowish colored colonies (see Supplementary Figure 4). Genes for carotenoid pigment production, giving the colony a yellowish color, were found on plasmid pPA_DBM3797_1, see Supplementary Table 5 and Supplementary Figure 1 (terpene).

**Table 3:** Ribonucleotide reductase [ribonucleoside diphosphate reductases (rNDP)] genes.

**Gene locus**	**Gene product annotation**	**Gene abbreviation**
**Ribonucleoside reductase class I (aerobic)**		
LKW31_04835	Class Ib ribonucleoside-diphosphate reductase subunit beta	*nrdF*
LKW31_04840	Class Ib ribonucleoside-diphosphate reductase subunit alpha	*nrdE*
LKW31_04845	Class Ib ribonucleoside-diphosphate reductase assembly flavoprotein NrdI	*nrdI*
LKW31_06395	Class I ribonucleotide reductase maintenance protein YfaE	*yfaE*
LKW31_06400	Class Ia ribonucleoside-diphosphate reductase subunit beta	*nrdB*
LKW31_06405	Class Ia ribonucleoside-diphosphate reductase subunit alpha	*nrdA*
**Ribonucleoside reductase (anaerobic)**		
LKW31_17070	Anaerobic ribonucleoside-triphosphate reductase	*nrdD*
LKW31_17075	Anaerobic ribonucleoside-triphosphate reductase-activating protein	*nrdG*

**Table 4:** Comparison of growth, acid and ethanol formation under aerobic or anaerobic conditions.

**Culture conditions**	**PGM aerobic**	**PGM anaerobic**	**LB with glucose, aerobic**	**LB with glucose, anaerobic**	**LB aerobic**
Lactic acid (g/l)	0.76 ± 0.12^b^	0.67 ± 0.11^b^	2.23 ± 0.17^c^	1.88 ± 0.05^c^	0.30 ± 0.02^a^
Acetic acid (g/l)	0.07 ± 0.01^a^	0.12 ± 0.01^b^	0.08 ± 0.01^a^	0.15 ± 0.01^b^	ND
Gluconic acid (g/l)	6.14 ± 0.12^b^	0.21 ± 0.02^a^	8.02 ± 0.20^c^	0.42 ± 0.07^a^	0.44 ± 0.12^a^
Ethanol (g/l)	0.35 ± 0.03^a^	0.27 ± 0.03^a^	0.34 ± 0.02^a^	0.35 ± 0.04^a^	ND
Cell dry weight (g/l)	1.10 ± 0.15^a^	0.90 ± 0.10^a^	2.80 ± 0.10^b^	2.50 ± 0.05^b^	3.70 ± 0.15^c^
pH	4.3 ± 0.1^b^	4.9 ± 0.1^c^	3.3 ± 0.1^a^	6.4 ± 0.1^d^	8.7 ± 0.1^e^

Glucose concentration in PGM and LB media with glucose was 10 g/l, culture experiments were performed in Erlenmeyer flasks in triplicate on a rotary shaker (aerobic condition) or in an anaerobic chamber (anaerobic conditions) for 48 h, and the pH of culture medium before inoculation was 6.8. ND, not detected; values labeled with identical letters are not significantly different at *p*-adjusted value < 0.05.

Under all conditions, acids were formed as the main primary metabolites, together with a small amount of ethanol. Acid formation resulted in a pH drop that caused growth to slow down and finally stop. While under anaerobic conditions, the main metabolites were acetic and lactic acids and ethanol, whereas under aerobic conditions, most of the glucose was oxidized to gluconic acid, plus the formation of lactic and acetic acids and ethanol. The concentration of lactic acid and the cell dry weight have rather significantly changed based on cultivation medium than based on aerobic/anaerobic conditions. However, statistically significant change based on aerobic/anaerobic conditions was observed for acetic acid and gluconic acid (with exception for LB aerobic cultivation condition). Ethanol level was similar under all conditions with no statistically significant change. Comparison of acid and ethanol production under different culture conditions is shown in Table 4 while the candidate genes coding for pyruvate processing into lactic and acetic acids and ethanol are shown in Table 5.

**Table 5:** Candidate genes for metabolite formation from pyruvate.

**Gene locus**	**Gene product annotation**	**Gene abbreviation**
**Lactic acid formation**		
LKW31_13490	D-lactate dehydrogenase	
LKW31_15200	FMN-dependent L-lactate dehydrogenase LldD	*lldD*
**Ethanol formation**		
LKW31_09010	Bifunctional acetaldehyde-CoA/alcohol dehydrogenase	*adhE*
**Acetate formation (anaerobic conditions)**		
LKW31_12905	Formate transporter FocA	
LKW31_12910	Formate C-acetyltransferase	*pfl*
LKW31_12915	Pyruvate formate lyase 1-activating protein	
LKW31_06270	Phosphate acetyltransferase	*pta*
LKW31_06275	Acetate kinase	*ack*

**Figure 3:**
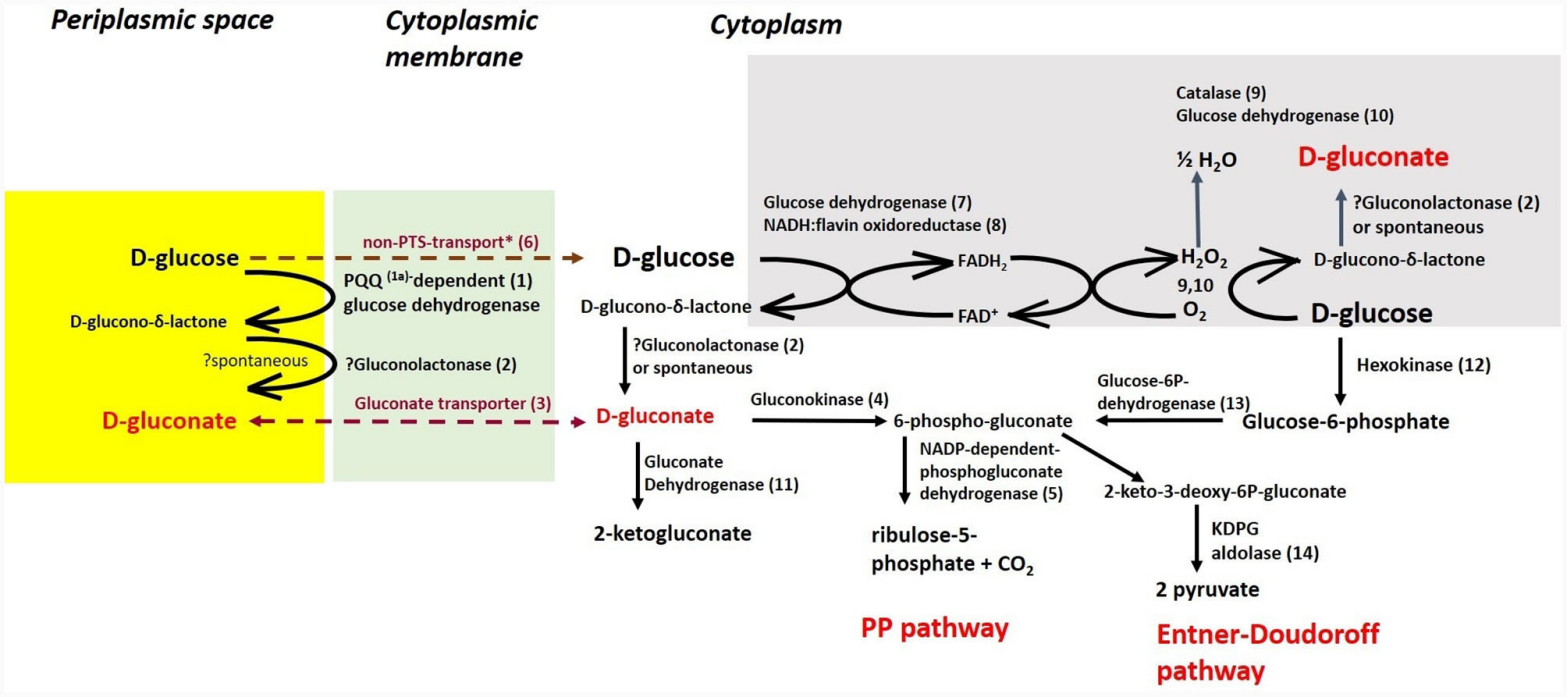
Putative pathways associated with gluconic acid production and its processing *in P. agglomerans* DBM 3797. The candidate genes for enzyme activities (1–14), as well as biosynthesis of pyrroloquinoline quinone (PQQ) (1a) are shown in Table 6. While the yellow highlighted part, which results in formation of extracellular gluconic acid, was confirmed by HPLC analysis, other proposed pathways require further confirmation. The cytoplasmic membrane is highlighted in green, intracellular gluconic acid production from glucose, atypical for bacteria, is highlighted in gray. PP stands for pentose phosphate. ^*^Stands for supposed glucose transport by passive diffusion.

Cultivations under different oxygen availability were also compared during bioreactor cultivations using PGM with 20 g/l of glucose (Supplementary Figure 5). Under aerobic conditions in a bioreactor, glucose consumption was double that under anaerobic conditions, but a substantial fraction of glucose was oxidized to gluconic acid. Oxygen limitation was observed during aerobic bioreactor culture, demonstrated as zero oxygen saturation from the 4th to the 14th hour of cultivation. The extracellular concentration of gluconic acid achieved was about 6 g/l in the bioreactor experiment using PGM and up to 8 g/l in shake flask experiments where LB medium supplemented with glucose (10 g/l) was used (see Table 5). A scheme demonstrating the putative gluconic acid metabolic pathways was created, see Figure 3, and respective candidate genes are shown in Table 6. While it seems that gluconic acid production is mostly mediated by membrane bound enzymes and is extracellular, gluconate can be transported into a bacterial cell by a specific gluconate transporter and this transport might be coupled with phosphorylation. The resulting 6-phospho-gluconate may be processed to metabolites entering either the Entner-Doudoroff or Pentose Phosphate pathways, see Figure 3.

**Table 6:** Candidate genes for gluconate metabolism found on the chromosome of *P. agglomerans* DBM 3797.

Gene locus^*^	**Gene product annotation**	**Gene abbreviation**
**PQQ dependent membrane bound glucose dehydrogenase (1)**		
LKW31_10030	Membrane-bound PQQ-dependent dehydrogenase, glucose/quinate/shikimate family	
LKW31_17685	Glucose/quinate/shikimate family membrane-bound PQQ-dependent dehydrogenase	
LKW31_05305	Glucose/quinate/shikimate family membrane-bound PQQ-dependent dehydrogenase	
**PQQ biosynthesis (1a)**		
LKW31_10225	Pyrroloquinoline quinone biosynthesis protein PqqF	*pqqF*
LKW31_10230	Pyrroloquinoline quinone biosynthesis protein PqqE	*pqqE*
LKW31_10235	Pyrroloquinoline quinone biosynthesis peptide PqqD	*pqqD*
LKW31_10240	Pyrroloquinoline-quinone synthase PqqC	*pqqC*
LKW31_10245	Pyrroloquinoline quinone biosynthesis peptide PqqB	*pqqB*
LKW31_10250	Pyrroloquinoline quinone precursor peptide PqqA	*pqqA*
**Gluconolactonase (2)**		
LKW31_05840	SMP-30/gluconolactonase/LRE family protein	
**Gluconate transport** + **phosphorylation (3,4)**		
LKW31_01595	Gluconate operon transcriptional repressor GntR	*gntR*
LKW31_01600	Gluconokinase	
LKW31_01605	Gluconate transporter	
**6-phosphogluconate dehydrogenase (5)**		
LKW31_07145	NADP-dependent phosphogluconate dehydrogenase	
**Glucose non-PTS transport (6)**		
LKW31_00160	Sugar ABC transporter permease	
**Glucose dehydrogenase (7)/NADH oxidoreductase (8)**		
LKW31_09960	Glucose 1-dehydrogenase	*gdh*
LKW31_09965	NADH:flavin oxidoreductase/NADH oxidase	
**Catalase (9)/glucose dehydrogenase (10)**		
LKW31_10630	Manganese catalase family protein	
LKW31_10635	Glucose 1-dehydrogenase	*gdh*
**Gluconate dehydrogenase (11)**		
LKW31_16920	Gluconate 2-dehydrogenase subunit 3 family protein	
LKW31_17770	Gluconate 2-dehydrogenase subunit 3 family protein	
**Hexokinase (12)**		
LKW31_05780	Glucokinase	*glk*
LKW31_10185	Glucokinase	
**13,14**		
LKW31_08485	MurR/RpiR family transcriptional regulator	
LKW31_08490	Glucose-6-phosphate dehydrogenase	
LKW31_08495	Bifunctional 4-hydroxy-2-oxoglutarate aldolase/2-dehydro-3-deoxy-phosphogluconate aldolase	
LKW31_02800	KDGP aldolase family protein	

^*^If there was a candidate transcriptional regulator in the vicinity of the candidate gene, it is shown too.

### 3.3 Plant growth promotion

Plant growth promoting activities were tested in a series of traditional microbiological assays and the candidate genes for all PGP activities are shown in Supplementary Table 5. There were confirmed high proteolytic/peptidase and cellulase activities, medium siderophore and IAA related compounds productions, weak amylolytic, lipolytic and pectinase activities. The ability to form indole acetic acid (IAA) or IAA-like compounds was tested in culture medium supplemented with the precursor compound, tryptophan and a positive reaction with Salkowski reagent was obtained. As the color was distinct compared to standard (IAA) as well as its retention time in UHPLC analysis (not shown), it is probable that not directly IAA, but a similar compound is formed. The most well-known gene of the IAA pathway, indolepyruvate decarboxylase, *ipdC*, was found in the genome (Supplementary Table 5). Further, symplasmata (biofilm like structure), carotenoid pigment formation and the ability to release ammonium mentioned above, can be considered PGP activities too. The ability to degrade ethylene was not tested, however the putative gene for 1-aminocyclopropane-1-carboxylate (ACC) deaminase was found on the chromosome. Phosphate solubilisation (PS) was tested in different types of tests but was not confirmed even if the genes for phosphonate metabolism and phosphate transporters were found in the genome (Supplementary Table 5) and gluconic acid formation was demonstrated.

## 4 Discussion

The *P. agglomerans* DBM 3797 strain isolated from fresh hop has a somewhat different phenotype from the similar strain *P. agglomerans* DBM 3796 isolated from dried hop (Kolek et al., 2021) and differed mainly in the low production of CO_2_ associated with low production of ethanol and acetic acid. The strain has two plasmids, whose circularity was proven during *de novo* assembly that produced circular contigs. Moreover, both plasmids contained the *repB* gene coding for plasmid replication initiator, suggesting that both plasmids formed integral parts of the *P. agglomerans* genome rather than foreign DNA. Additionally, no intact prophage sequences were found on plasmids. The first plasmid pPA_DBM3797_1, of size 555 kb and harboring genes for carotenoid biosynthesis and siderophores (Supplementary Table 5), as well as thiamine biosynthesis (not shown) genes; these seem to meet the criteria for a large universal *Pantoea* plasmid (De Maayer et al., 2012). Growth under both aerobic and anaerobic conditions correlates with the possibility of synthesizing deoxyribonucleotides for DNA replication during growth by class I (aerobic) and class III (anaerobic) ribonucleotide reductases (Torrents, 2014). The second plasmid, pPA_DBM3797_2 is, according to a functional annotation, responsible for signal transduction and defense mechanisms rather than metabolism and carries one gene responsible for antibiotic resistance from pmr phosphoethanolamine transferase gene family. This gene might be involved in polymyxin resistance (Huang et al., 2018) and there is a potential risk for its spreading by horizontal gene transfer. Nevertheless, the risk assessment requires further study. Other antibiotic resistance genes are of lower risk as they are located on chromosome and in addition, a lot of them are efflux pumps genes which might be attributed to the need to resist the action of antimicrobial substances produced by the host hop plant. The antimicrobial active substances of hops include, for example, beta-acids, effective against methicillin resistant *Staphylococcus aureus* strains (Sleha et al., 2021). The absence of a native CRISPR-Cas system that can serve as a form of bacterial immunity (Sorek et al., 2013) is compensated for by the presence of numerous R-M systems. At least some of these systems are probably active, as *P. agglomerans* DBM 3797 contains only a minimum of foreign DNA, particularly only one intact prophage PHAGE_Erwini_ENT90_NC_019932. The presence of such foreign DNA is not unique for *P. agglomerans* as the very same prophage was previously identified in the genome of the strain *P. agglomerans* C1 (Luziatelli et al., 2019).

The most closely related strains, AB378 and CFBP8784, were, like strain DBM 3797, isolated from the phyllosphere of plants, specifically from Red Topaz apple blossom (AB378) and from radish *Raphanus sativus* flower (CFBP8784). Together with other neighboring strains (Figure 2), i.e., Pan8 (isolated from *Pisum sativum* phyllosphere), P10c (isolated from apple tree), and DOAB1048 (isolated from wheat leaves) they form a group of *P. agglomerans* environmental strains isolated from above-ground plant parts and differ from strains P5, ANP8, CPHN2, and DAPP-PG744 (Table 1) isolated from plant roots or from soil.

The ability to form a biofilm is considered to be an advantage for the bacteria colonizing the plants, as the biofilm protects both the bacterial population from adverse environmental influences and the colonized plant surface. In addition, the ability to communicate between the microbial community and the plant cells is enhanced by signal amplification during biofilm formation (Seneviratne et al., 2010). Symplasmata i.e., multicellular round aggregates mimicking colonies in a liquid medium, probably gave the original name to the species “agglomerans” (Tecon and Leveau, 2016) and were described in detail e.g., in the rice epiphyte *P. agglomerans* YS19 (Yu et al., 2016; Zheng et al., 2019). Although these formations were firstly described by M. W. Beijerinck in 1888 (Tecon and Leveau, 2016), it is still not completely clear why they are formed. They provide protection to the bacteria, comparable to biofilm formation, and are considered to be an advantage for colonization of rice roots (Achouak et al., 1994). Symplasmata formation seems to be initiated by culture conditions but different reports differ in descriptions of which factor is decisive. While *Pantoea eucalypti* best formed symplasmata in glucose medium not in LB medium, and at a pH higher than 7.6 (Tecon and Leveau, 2016), our strain *P. agglomerans* DBM 3797 formed them only in LB medium at pH 8.5. Our findings are in accordance with those for strain YS19 (Jiang et al., 2015; Yu et al., 2016; Zheng et al., 2019) and nitrogen-fixing *E. agglomerans* NO30 (Achouak et al., 1994). Symplasmata of strain YS19 were probably regulated by indole (Yu et al., 2016) and it was hypothesized that indole originated from tryptophan degradation and was considered by the *P. agglomerans* indolenon-producing strain as a marker of starvation (Jia et al., 2017). In addition, in the same strain YS19, an acyl-homoserine lactone quorum sensing system was also involved in symplasmata formation (Jiang et al., 2015). While the genes for acyl-homoserine lactone were confirmed in our strain by antiSMASH analysis, indole release was not confirmed. Symplasmata formation was also found in an anaerogenic group of *Enterobacter* clinical isolates (Gilardi and Bottone, 1971), which corresponds to the finding that genes for anaerobic metabolism were active during symplasmata formation in *P. eucalypti* aerobic culture (Tecon and Leveau, 2016) and it was deduced that there is an oxygen limitation inside these formations. The ability to cope with anaerobic conditions was proven in our strain.

The ability to utilize peptides in LB medium corresponds with the number of peptidase genes present in the genome (not shown). This type of fermentation is typical for certain fermented foods such as natto (fermented soybeans) or pidan (fermented eggs) rich in proteins and peptides (Wang and Fung, 1996) and was also described in detail for *E. coli* (Sezonov et al., 2007). Aerobic growth in LB medium is a special type of alkaline fermentation where amino acids are used as carbon sources instead of saccharides. Generally, it is supposed that amino acids are processed by oxidative deamination, generating α-keto acids and ammonia. It is believed that the key role in the process is played by glutamate dehydrogenase, but its respective candidate gene was not found in the genome. Thus, it seems more likely that metabolism of each amino acid is unique and while the ammonium cation is released or transferred to other compounds, the rest of the molecule can be transformed to pyruvate, acetyl CoA, acetoacetyl CoA and intermediates of the citric acid cycle under aerobic conditions, as described by Li et al. (2018). Part of the ammonium ion is used by the bacterium but its release is excessive which results in an increase in pH. Actually, the release of ammonium ions from available amino acids and peptides can be one of the advantageous features of the DBM 3797 strain, which might be used for controlled ammonia release if the strain was applied together with organic fertilizers.

In many aerobic Gram-negative bacteria, gluconic acid is formed by glucose dehydrogenase through D-glucono-δ-lactone in the periplasmic space (Ma et al., 2022). Further, it is expected that transport of gluconate from the periplasmic space through the outer membrane is mediated by porins. Based on knowledge gathered for *Gluconobacter oxydans* (Pronk et al., 1989), extracellular gluconic acid production is probably a result of the membrane bound PQQ-dependent glucose dehydrogenase, which was described in detail for *Pantoea ananatis* (Andreeva et al., 2011). Candidate genes for this enzyme activity, as well as the complete *pqqABCDEF* biosynthetic operon, were found in the genome. In Gram-negative aerobic bacteria, there are frequently found other membrane bound enzymes, such as PQQ-dependent 5-keto-gluconate dehydrogenase, and flavin/heme dependent gluconate and 2-keto-gluconate dehydrogenases (Ma et al., 2022), but the candidate genes were not found in the genome. Microbial gluconic acid production was reviewed by Ramachandran et al. (2006) and Ma et al. (2022) and it is obvious that fungi and bacteria differ in metabolic pathways for gluconic acid production. In fungi, such as in *Aspergillus niger*, its main industrial producer, gluconic acid is produced by FAD^+^-dependent glucose oxidase, which is coupled with catalase (Ramachandran et al., 2006) and surprisingly, similar candidate genes coding for this option, which are atypical for bacteria, were also found in our genome (see Figure 3; Table 5). Further, it appears that the catabolic pathway of glucose in the studied strain may use parts of known metabolic pathways such as the Entner-Doudoroff, Embden-Meyer-Parnas or Pentose Phosphate pathways, which are interconnected, similar to what has been found and described for *Pseudomonas putida* KT2440 (Nikel et al., 2015).

Gluconic acid production may be associated with defense against soil protozoa such as *Vahlkampfia* sp. or *Neobodo designis* (Gómez et al., 2010), however mostly it is exploited together with other organic acids in the solubilization of inorganic phosphate. Phosphate solubilization is a significant PGP feature that facilitates plant growth by increasing its accessibility from both inorganic and organic phosphate-containing compounds and complexes (Rawat et al., 2021). Typical phosphate solubilizing microorganisms, in addition to the formation of organic acids, may form siderophores, exopolysaccharides, phosphatases, phosphonatases and others (Liang et al., 2020; Rawat et al., 2021), and the genes for these functions were found in our strain too but actual PS activity was not confirmed in any type of applied test. Siderophore as well as metalophore formation gene clusters were also identified by antiSMASH. This finding is consistent with recently published information (Elhaissoufi et al., 2023) that even bacteria not showing PS capability in a given test can in fact contribute significantly to plant phosphorus supply.

The thiopeptide formation ability revealed by the antiSMASH analysis (Supplementary Figure 1) may indicate microcin(s), bioactive peptide(s) production [namely class IIa microcin having disulphide bond(s) in their structure (Parker and Davies, 2022)]. Microcin production was confirmed in *P. agglomerans* Eh252 (Vanneste et al., 2002) and the *P. agglomerans* E325 producing microcins was even applied for biocontrol of fire blight disease of apple, caused by *Erwinia amylovora* (Kim et al., 2012).

Aerobic metabolism of the strain is also associated with the production of potential auxin compounds, IAA-like substances, in the culture medium supplemented with tryptophan. In *P. agglomerans*, IAA biosynthesis begins with the formation of indole-3-pyruvic acid, mediated by aminotransferase, continues with indole-3-acetaldehyde formation by indolepyruvate decarboxylase, coded by *ipdC*, and ends with IAA formation catalyzed by indole-3-acetaldehyde dehydrogenase (Luziatelli et al., 2020b). In our strain, the *ipdC* gene was found, several candidate aminotransferase genes (not shown), but not the gene for indole-3-acetaldehyde dehydrogenase. Since the traditional method for detection of IAA using the Salkowski reagent resulted in the formation of an orange color with an absorption maximum of 450 nm rather than a pink color with a maximum of 530 nm, indole-3-butyric acid (IBA) was tested as a possible previously described (Gilbert et al., 2018) product of this reaction. Unfortunately, IBA was not confirmed as the reaction product. Gilbert et al. (2018) demonstrated that different bacterial isolates produced different compounds with potential auxin activity from tryptophan and we concluded that our strain probably belongs to this IAA-like compound producers' group.

*P. agglomerans*, strain DBM 3797, isolated from hops has a number of properties potentially beneficial to the hop plant, but its safety profile needs to be addressed in follow-up research. In particular, the possibility of horizontal transfer of antibiotic resistance genes, which has been little studied in the genus *Pantoea*, and virulence genes that may lead to pathogenicity in plants or animals, and humans in some strains of the species (Guevarra et al., 2021), need to be focused on. Unfortunately, there are not enough complete genome assemblies yet for a detailed comparison of particular strains. Although there are some specific inserts in the genome of *P. agglomerans* DBM 3797 in comparison to additional five strains (Supplementary Figure 2), no specific feature distinguishing pathogens from harmless strains isolated from above-ground parts of plants.

## Data availability statement

The whole-genome sequence and plasmid sequences were deposited in the DDBJ/ENA/GenBank under accession numbers CP086133.1, CP086134.1, and CP086135.1, respectively. The NCBI BioProject and BioSample IDs are PRJNA774971 and SAMN22600026. The raw reads were deposited in the NCBI SRA database under accession numbers SRR25382413 (paired-end Illumina) and SRR25382412 (Oxford Nanopore Technologies).

## Author contributions

PP: Conceptualization, Funding acquisition, Writing – original draft, Writing – review & editing. MV: Investigation, Writing – review & editing. KS: Conceptualization, Data curation, Funding acquisition, Writing – original draft, Writing – review & editing. KJ: Investigation, Visualization, Writing – review & editing. MB: Formal analysis, Investigation, Writing – review & editing. PL: Investigation, Writing – review & editing. BB: Investigation, Writing – review & editing. PK: Funding acquisition, Writing – review & editing. KK: Conceptualization, Investigation, Writing – review & editing.

## Footnotes

^1^NIH, National Center for Biotechnology Information (2024). Available online at: https://www.ncbi.nlm.nih.gov/datasets/genome/?taxon=549&annotated_only=true&refseq_annotation=true&genbank_annotation=true&assembly_level=3%3A3.

## References

[B1] Achouak W. Heulin T. Villemin G. J. Balandreau J. (1994). Root colonization by symplasmata-forming *Enterobacter agglomerans*. FEMS Microbiol. Ecol. 13, 287–294. 10.1111/j.1574-6941.1994.tb00075.x

[B2] Alcock B. P. Raphenya A. R. Lau T. T. Y. Tsang K. K. Bouchard M. Edalatmand A.. (2020). CARD 2020: antibiotic resistome surveillance with the comprehensive antibiotic resistance database. Nucleic Acids Res. 48, D517–D525. 10.1093/nar/gkz935 31665441 PMC7145624

[B3] Alikhan N. F. Petty N. K. Ben Zakour N. L. Beatson S. A. (2011). BLAST Ring Image Generator (BRIG): simple prokaryote genome comparisons. BMC Genom. 12:402. 10.1186/1471-2164-12-402 21824423 PMC3163573

[B4] Allen M. E. Piefer A. J. Cole S. N. Werner J. J. Benziger P. T. Grieneisen L.. (2019). Characterization of microbial communities populating the inflorescences of *Humulus lupulus* L. J. Am. Soc. Brew. Chem. 77, 243–250. 10.1080/03610470.2019.1667739

[B5] Altschul S. F. Gish W. Miller W. Myers E. W. Lipman D. J. (1990). Basic local alignment search tool. J. Mol. Biol. 215, 403–410. 10.1016/S0022-2836(05)80360-2 2231712

[B6] Andreeva I. G. Golubeva L. I. Kuvaeva T. M. Gak E. R. Katashkina J. I. Mashko S. V. (2011). Identification of *Pantoea ananatis* gene encoding membrane pyrroloquinoline quinone (PQQ)-dependent glucose dehydrogenase and *pqqABCDEF* operon essential for PQQ biosynthesis. FEMS Microbiol. Lett. 318, 55–60. 10.1111/j.1574-6968.2011.02240.x 21306430

[B7] Arndt D. Grant J. Marcu A. Sajed T. Pon A. Liang Y.. (2016). PHASTER: a better, faster version of the PHAST phage search tool. Nucleic Acids Res. 44, W16–W21. 10.1093/nar/gkw387 27141966 PMC4987931

[B8] Biswas A. Staals R. H. J. Morales S. E. Fineran P. C. Brown C. M. (2016). CRISPRDetect: a flexible algorithm to define CRISPR arrays' *BMC Genom*. 17:356. 10.1186/s12864-016-2627-0 27184979 PMC4869251

[B9] Blin K. Shaw S. Augustijn H. E. Reitz Z. L. Biermann F. Alanjary M.. (2023). antiSMASH 7.0: new and improved predictions for detection, regulation, chemical structures and visualisation. Nucl. Acids Res. 51. W46–W50. 10.1093/nar/gkad344 37140036 PMC10320115

[B10] Bocquet L. Sahpaz S. Rivière C. (2018). “An overview of the antimicrobial properties of hop,” in Natural Antimicrobial Agents. Sustainable Development and Biodiversity, Vol 19, eds J. M. Mérillon, and C. Riviere (Cham: Springer).

[B11] Bolger A. M. Lohse M. Usadel B. (2014). Trimmomatic: a flexible trimmer for Illumina sequence data. Bioinformatics 30, 2114–2120. 10.1093/bioinformatics/btu170 24695404 PMC4103590

[B12] Cantalapiedra C. P. Hernández-Plaza A. Letunic I. Bork P. Huerta-Cepas J. (2021). EggNOG-Mapper v2: functional annotation, orthology assignments, and domain prediction at the metagenomic scale. Mol. Biol. Evol. 38, 5825–5829. 10.1093/molbev/msab293 34597405 PMC8662613

[B13] Carver T. Harris S. H. Berriman M. Parkhill J. McQuillan J. A. (2012). Artemis: an integrated platform for visualization and analysis of high-throughput sequence-based experimental data. Bioinformatics 28, 464–469. 10.1093/bioinformatics/btr703 22199388 PMC3278759

[B14] Carver T. Thomson N. Bleasby A. Berriman M. Parkhill J. (2009). DNAPlotter: circular and linear interactive genome visualization. Bioinformatics 25, 119–120. 10.1093/bioinformatics/btn578 18990721 PMC2612626

[B15] Chaudhari N. M. Gupta V. K. Dutta C. (2016). BPGA-an ultra-fast pan-genome analysis pipeline. Sci. Rep. 6:24373. 10.1038/srep24373 27071527 PMC4829868

[B16] De Maayer P. Chan W. Y. Blom J. Venter S. N. Duffy B. Smits T. H. M.. (2012). The large universal *Pantoea* plasmid LPP-1 plays a major role in biological and ecological diversification. BMC Genom. 13:625. 10.1186/1471-2164-13-625 23151240 PMC3505739

[B17] Dutkiewicz J. Mackiewicz B. Lemieszek M. K. Golec M. Milanowski J. (2016). *Pantoea agglomerans*: a mysterious bacterium of evil and good. Part IV. Beneficial effects. Ann. Agric. Environ. Med. 23, 206–222. 10.5604/12321966.1203879 27294621

[B18] Edgar R. C. (2010). Search and clustering orders of magnitude faster than BLAST. Bioinformatics 26, 2460–2461. 10.1093/bioinformatics/btq461 20709691

[B19] Elhaissoufi W. Ibnyasser A. Haddine M. Zeroual Y. Ghani R. Barakat A.. (2023). Screening of potential phosphate solubilizing bacteria inoculants should consider the contrast in phosphorus bio-solubilization rate along with plant growth promotion and phosphorus use efficiency. J. Appl. Microbiol. 134:lxac077. 10.1093/jambio/lxac077 36724266

[B20] Ewels P. Magnusson. M Lundin. S Käller M. (2016). MultiQC: summarize analysis results for multiple tools and samples in a single report. Bioinformatics 32, 3047–3048. 10.1093/bioinformatics/btw354 27312411 PMC5039924

[B21] Fahle A. Bereswill S. Heimesaat M. M. (2022). Antibacterial effects of biologically active ingredients in hop provide promising options to fight infections by pathogens including multi-drug resistant bacteria. Eur. J. Microbiol. Immunol. 12, 22–30. 10.1556/1886.2022.00006 35417405 PMC9036650

[B22] Gilardi G. L. Bottone E. (1971). *Erwinia* and yellow-pigmented *Enterobacter* isolates from human sources. Antonie Van Leeuwenh. 37, 529–535. 10.1007/BF02218523 5316523

[B23] Gilbert S. Xu J. Acosta K. Poulev A. Lebeis S. Lam E. (2018). Bacterial production of indole related compounds reveals their role in association between duckweeds and endophytes. Front. Chem. 6:265. 10.3389/fchem.2018.00265 30050896 PMC6052042

[B24] Gómez W. Buela L. Castro L. T. Chaparro V. Ball M. M. Yarzábal L. A. (2010). Evidence for gluconic acid production by *Enterobacter intermedium* as an efficient strategy to avoid protozoan grazing. Soil Biol. Biochem. 42, 822–830. 10.1016/j.soilbio.2010.01.019

[B25] Goryluk-Salmonovicz A. Piórek M. Rekosz-Burlaga H. Studnicki M. Blasczyk M. (2016). Endophytic detection in selected European herbal plants. Polish J. Microbiol. 65, 369–375. 10.5604/17331331.1215617 29334055

[B26] Guevarra R. B. Magez S. Peeters E. Chung M. S. Kim K. H. Radwanska M. (2021). Comprehensive genomic analysis reveals virulence factors and antibiotic resistance genes in *Pantoea agglomerans* KM1, a potential opportunistic pathogen. PLoS ONE 16:e0239792. 10.1371/journal.pone.0239792 33406073 PMC7787473

[B27] Hawar S. N. (2022). Extracellular enzyme of endophytic fungi isolated from *Ziziphus spina* leaves as medicinal plant. Int. J. Biomater. 2022:2135927. 10.1155/2022/2135927 35845475 PMC9279100

[B28] Huang J. Zhu Y. Han M. L. Li M. Song J. Velkov T.. (2018). Comparative analysis of phosphoethanolamine transferases involved in polymyxin resistance across 10 clinically relevant Gram-negative bacteria. Int. J. Antimicrob. Agents 51, 586–593. 10.1016/j.ijantimicag.2017.12.016 29288722 PMC5869126

[B29] Jaskula B. Kafarski P. Aerts G. De Cooman L. (2008). A kinetic study on the isomerization of hop alpha acids. J. Agric. Food Chem. 56, 6408–6415. 10.1021/jf8004965 18598038

[B30] Jia M. Yu X. Jiang J. Li Z. Feng Y. (2017). The cytidine repressor participates in the regulatory pathway of indole in *Pantoea agglomerans*. Res. Microbiol. 7, 636–643. 10.1016/j.resmic.2017.04.006 28483441

[B31] Jiang J. Wu S. Wang J. Feng Y. (2015). AHL-type quorum sensing and its regulation on symplasmata formation in *Pantoea agglomerans* YS19. J. Basic Microbiol. 55, 607–616. 10.1002/jobm.201400472 25283544

[B32] Kim I. J. Pusey P. L. Zhao Y. Korban S. S. Choi H. Kim K. K. (2012). Controlled release of *Pantoea agglomerans* E325 for biocontrol of fire blight disease of apple. J. Controll. Release 161, 109–115. 10.1016/j.jconrel.2012.03.028 22516094

[B33] Koçak O. F. (2019). Identification of *Streptomyces* strains isolated from *Humulus lupulus* rhizosphere and determination of plant growth promotion potential of selected strains. Turk. J. Biol. 43, 391–403. 10.3906/biy-1906-37 31892814 PMC6911257

[B34] Kolek J. Patakova P. Junkova P. Krofta K. Hynek R. Dostalek P. (2021). Isolation and identification of *Pantoea agglomerans* from the inflated bag with dried hop pellets stored under a modified atmosphere. J. Appl. Microbiol. 131, 281–287. 10.1111/jam.14970 33320407

[B35] Kumar P. Rani S. Dahiya P. Kumar A. Dang A. S. Pooja S. (2022). Whole genome analysis for plant growth promotion profiling of *Pantoea agglomerans* CPHN2, a non-rhizobial nodule endophyte. Front. Microbiol. 13:998821. 10.3389/fmicb.2022.998821 36419432 PMC9676466

[B36] Lanfear R. Schalamun M. Kainer D. Wang W. Schwessinger B. (2019). MinIONQC: fast and simple quality control for MinION sequencing data. Bioinformatics 35, 523–525. 10.1093/bioinformatics/bty654 30052755 PMC6361240

[B37] Li H. (2018). Minimap2: pairwise alignment for nucleotide sequences. Bioinformatics 34, 3094–3100. 10.1093/bioinformatics/bty191 29750242 PMC6137996

[B38] Li H. Durbin R. (2009). Fast and accurate short read alignment with Burrows–Wheeler transform. Bioinformatics 25, 1754–1760. 10.1093/bioinformatics/btp324 19451168 PMC2705234

[B39] Li H. Handsaker B. Wysoker A. Fennel,l T. Ruan J. Homer N.. (2009). The sequence alignment/map format and SAMtools. Bioinformatics 25, 2078–2079. 10.1093/bioinformatics/btp352 19505943 PMC2723002

[B40] Li S. Y. Ng I. S. Chen P. T. Chiang C. J. Chao Y. P. (2018). Biorefining of protein waste for production of sustainable fuels and chemicals. Biotechnol. Biofuels 11:256. 10.1186/s13068-018-1234-5 30250508 PMC6146663

[B41] Liang J. L. Liu J. Jia P. Yang T. T. Zeng Q. W.. (2020). Novel phosphate-solubilizing bacteria enhance soil phosphorus cycling following ecological restoration of land degraded by mining. ISME J. 14, 1600–1613. 10.1038/s41396-020-0632-4 32203124 PMC7242446

[B42] Liu B. Zheng D. Jin Q. Chen L. Yang J. (2019). VFDB 2019: a comparative pathogenomic platform with an interactive web interface. Nucl. Acids Res. 47, D687–D692. 10.1093/nar/gky1080 30395255 PMC6324032

[B43] Luo H. Quan C.-L. Peng C. Gao F. (2019). Recent development of Ori-Finder system and DoriC database for microbial replication origins. Brief. Bioinform. 20, 1114–1124. 10.1093/bib/bbx174 29329409

[B44] Luziatelli F. Ficca A. G. Bonini P. Muleo R. Gatti L. Meneghin.i M. Tronati M. Melini F. Ruzzi M (2020b). Genetic and metabolomic perspective on the production of indole-3-acetic acid by *Pantoea agglomerans* and use of their metabolites as biostimulants in plant nurseries. Front. Microbiol. 11:1475. 10.3389/fmicb.2020.01475 32765438 PMC7381177

[B45] Luziatelli F. Ficca A. G. Cardarelli M. Melini F. Cavalieri A. Ruzzi M. (2020a). Genome sequencing of *Pantoea agglomerans* C1 provides insights into molecular and genetic mechanisms of plant growth-promotion and tolerance to heavy metals. Microorganisms 8:153. 10.3390/microorganisms8020153 31979031 PMC7074716

[B46] Luziatelli F. Ficca A. G. Melini F. Ruzzi M. (2019). Genome sequence of the plant growth-promoting rhizobacterium *Pantoea agglomerans* C1. Microbiol. Resour. Announ. 8, e00828–e00819. 10.1128/MRA.00828-19 31672740 PMC6953516

[B47] Ma Y. Li B. Zhang X. Wang C. Chen W. (2022). Production of gluconic acid and its derivatives by microbial fermentation: Process improvement based on integrated routes. Front. Bioeng. Biotechnol. 10:864787. 10.3389/fbioe.2022.864787 35651548 PMC9149244

[B48] Marchler-Bauer A. Bryant S. H. (2004). CD-search: protein domain annotations on the fly. Nucl. Acids Res. 32, W327–W331. 10.1093/nar/gkh454 15215404 PMC441592

[B49] Micci A. Zhang Q. Chang X. Kingsley K. Park L. Chiaranunt P.. (2022). Histochemical evidence for nitrogen-transfer endosymbiosis in non-photosynthetic cells of leaves and inflorescence bracts of angiosperms. Biology 11:876. 10.3390/biology11060876 35741397 PMC9220352

[B50] Milligan S. R. Kalita J. C. Pocock V. Van de Kauter V. Stevens J. F. Deinzer M. L.. (2000). The endocrine activities of 8-prenylnaringenin and related hop (*Humulus lupulus* L.) flavonoids. J. Clin. Endocrinol. Metab. 85, 4912–4915. 10.1210/jcem.85.12.7168 11134162

[B51] Miranda C. L. Stevens J. F. Helmrich A. Henderson M. C. Rodriguez R. J. Yang Y. H.. (1999). Antiproliferative and cytotoxic effects of prenylated flavonoids from hops (*Humulus lupulus*) in human cancer cell lines. Food Chem. Toxicol. 37, 271–285. 10.1016/S.0278-6915(99)00019-8 10418944

[B52] Nikel P. I. Chavarría M. Fuhrer T. Sauer U. de Lorenzo V. (2015). *Pseudomonas putida* KT2440 strain metabolizes glucose through a cycle formed by enzymes of the Entner-Doudoroff, Embden-Meyerhof-Parnas, and pentose phosphate pathways. J. Biol. Chem. 290, 25920–25932. 10.1074/jbc.M115.687749 26350459 PMC4646247

[B53] Noori F. Etesami H. Noori S. Forouzan E. Jouzani G. S. Malboobi M. A. (2021). Whole genome sequence of *Pantoea agglomerans* ANP8, a salinity and drought stress–resistant bacterium isolated from alfalfa (*Medicago sativa* L.) root nodules. Biotechnol. Rep. 29:e00600. 10.1016/j.btre.2021.e00600 33643858 PMC7893418

[B54] O'Leary N. A. Wright M. W. Brister J. R. Ciufo S. Haddad D. McVeigh R.. (2016). Reference sequence (RefSeq) database at NCBI: current status, taxonomic expansion, and functional annotation. Nucl. Acids Res. 44, D733–745. 10.1093/nar/gkv1189 26553804 PMC4702849

[B55] Parker J. K. Davies B. W. (2022). Microcins reveal natural mechanisms of bacterial manipulation to inform therapeutic development. Microbiology 168:001175. 10.1099/mic.0.001175 35438625 PMC10233263

[B56] Pronk J. T. Levering P. R. Olijve W. van Dijken J. P. (1989). Role of NADP-dependent and quinoprotein glucose dehydrogenases in gluconic acid production by *Gluconobacter oxydans*. Enzyme Microb. Technol. 11, 160–164. 10.1016/0141-0229(89)90075-6

[B57] Ramachandran S. Fontanille P. Pandey A. Larroche C. (2006). Gluconic acid: properties, applications and microbial production. Food Technol. Biotechnol. 44, 185–195. Available online at: https://hrcak.srce.hr/file/161891

[B58] Rawat P. Das S. Shankhdhar D. Shankhdar S. C. (2021). Phosphate-solubilizing microorganisms: mechanism and their role in phosphate solubilization and uptake. J. Soil Sci. Plant Nutr. 21, 49–68. 10.1007/s42729-020-00342-7

[B59] Rezzonico F. Smits T. H. Montesinos E. Frey J. E. Duffy B. (2009). Genotypic comparison of *Pantoea agglomerans* plant and clinical strains. BMC Microbiol. 9:204. 10.1186/1471-2180-9-204 19772624 PMC2764716

[B60] Roberts R. J. Vincze T. Posfai J. Macelis D. (2023). REBASE: a database for DNA restriction and modification: enzymes, genes and genomes. Nucl. Acids Res. 51, D629–D630. 10.1093/nar/gkac975 36318248 PMC9825431

[B61] Schmidt C. S. Lovecka P. Mrnka L. Vychodilova A. Strejcek M. Fenclova M.. (2018). Distinct communities of poplar endophytes on an unpolluted and a risk element-polluted site and their plant growth-promoting potential *in vitro*. Microb. Ecol. 75, 955–969. 10.1007/s00248-017-1103-y 29127500

[B62] Sedlar K. Vasylkivska M. Musilova J. Branska B. Provaznik I. Patakova P. (2021). Phenotypic and genomic analysis of isopropanol and 1,3-propanediol producer *Clostridium diolis* DSM 15410. Genomics 113, 1109–1119. 10.1016/j.ygeno.2020.11.007 33166602

[B63] Seneviratne G. Weerasekara M. L. M. A.W. Seneviratne C. Zavahir J. S. Kcskés M. L. Kennedy I. R. (2010). “Importance of biofilm formation in plant growth promoting rhizobacterial action,” in Plant Growth and Health Promoting Bacteria, ed D. K. Maheshawari (Berlin: Springer-Verlag), 81–95.

[B64] Sevigny J. L. Lloyd B. McComish C. Ramsey A. Koziol L. (2019). Whole-genome sequences of *Pantoea agglomerans* BL3, *Pseudomonas fluorescens* BL, and *Pseudomonas stutzeri* CM14, isolated from Hops (*Humulus lupulus*). Microbiol. Resour. Announ. 8, e00545–e00519. 10.1128/MRA.00545-19 31346016 PMC6658686

[B65] Sezonov G. Joseleau-Petit D. D'Ari R. (2007). *Escherichia coli* physiology in Luria-Bertani broth. J. Bacteriol. 189, 8746–8749. 10.1128/JB.01368-07 17905994 PMC2168924

[B66] Shariati J. V Malboobi M. A. Tabrizi Z. Tevakol E. Owlia P.. (2017). Comprehensive genomic analysis of a plant growth-promoting rhizobacterium *Pantoea agglomerans* strain P5. Sci. Rep. 7:15610. 10.1038/s41598-017-15820-9 29142289 PMC5688152

[B67] Sleha R. Radochova V. Malis J. Mikyska A. Houska M. Krofta K.. (2021). Strong antimicrobial and healing effects of Beta-Acids from Hops in methicillin-resistant *Staphylococcus aureus*-infected external wounds *in vivo*. Antibiotics 10:708. 10.3390/antibiotics10060708 34204644 PMC8231114

[B68] Sorek R. Lawrence C. M. Wiedenheft B. (2013). CRISPR-mediated adaptive immune systems in bacteria and archaea. Annu. Rev. Biochem. 82, 237–266. 10.1146/annurev-biochem-072911-172315 23495939

[B69] Soutar C. D. Stavrinides J. (2019). Molecular validation of clinical *Pantoea* isolates identified by MALDI-TOF. PLoS ONE 14:e0224731. 10.1371/journal.pone.0224731 31682625 PMC6827907

[B70] Stranska M. Lovecka P. Vrchotova B. Uttl L. Bechynska K. Behner A.. (2021). Bacterial endophytes from *Vitis vinifera* L – Metabolomics characterization of plant-endophyte crosstalk. Chem. Biodivers. 18:e2100516. 10.1002/cbdv.202100516 34609783

[B71] Sulja A. Pothier J. F. Blom J. Moretti C. Buonario R. Rezzonico F.. (2022). Comparative genomics to examine the endophytic potential of *Pantoea agglomerans* DAPP-PG 734. BMC Genom. 23:742. 10.1186/s12864-022-08966-y 36344949 PMC9641835

[B72] Taboada B. Estrada K. Ciria R. Merino E. (2018). Operon-mapper: a web server for precise operon identification in bacterial and archaeal genomes. Bioinformatics 34, 118–120. 10.1093/bioinformatics/bty496 29931111 PMC6247939

[B73] Tatusova T. DiCuccio M. Badretdin A. Chetvernin V. Nawrocki E. P. Zaslavsky L.. (2016). NCBI prokaryotic genome annotation pipeline. Nucl. Acids Res. 44, 6614–6624. 10.1093/nar/gkw569 27342282 PMC5001611

[B74] Tecon R. Leveau J. (2016). Symplasmata are a clonal, conditional, and reversible type of bacterial multicellularity. Sci. Rep. 6:31914. 10.1038/srep31914 27534795 PMC4989142

[B75] Torrents E. (2014). Ribonucleotide reductases: essential enzymes for bacterial life. Front. Cell. Infect. Microbiol. 4:52. 10.3389/fcimb.2014.00052 24809024 PMC4009431

[B76] Vanneste J. L. Cornish D. A. Yu J. Voyle M. D. (2002). The peptide antibiotic produced by *Pantoea agglomerans* EH252 is a microcin. Acta Hortic. 590, 285–290. 10.17660/ActaHortic.2002.590.42

[B77] Vaser R. Sović I. Nagarajan N. Šikić M (2017). Fast and accurate *de novo* genome assembly from long uncorrected reads. Genome Res. 27, 737–746. 10.1101/gr.214270.116 28100585 PMC5411768

[B78] Walker B. J. Abeel T. Shea T. Priest M. Abouellie,l A. Sakthikumar S.. (2014). Pilon: an integrated tool for comprehensive microbial variant detection and genome assembly improvement. PLoS ONE 9:e112963. 10.1371/journal.pone.0112963 25409509 PMC4237348

[B79] Walterson A. M. Stavrinides J. (2015). *Pantoea:* insights into a highly versatile and diverse genus within the Enterobacteriaceae. FEMS Microbiol. Rev. 39, 968–984. 10.1093/femsre/fuv027 26109597

[B80] Wang J. Fung D. Y. C. (1996). Alkaline-fermented foods: a review with emphasis on pidan fermentation. Crit. Rev. Microbiol. 2, 101–138. 10.3109/10408419609106457 8817079

[B81] Yu X. Jiang J. Liang C. Zhang X. Wang J. Shen D.. (2016). Indole affects the formation of multicellular aggregate structures in *Pantoea agglomerans* YS19. J. Gen. Appl. Microbiol. 62, 31–37. 10.2323/jgam.62.31 26923129

[B82] Zhang S. Soltis D. E. Yang Y. Li D. Yi T. (2011). Multi-gene analysis provides a well-supported phylogeny of Rosales. Mol. Phylogenet. Evol. 60, 21–28. 10.1016/j.ympev.2011.04.008 21540119

[B83] Zheng J. Xia Y. Liu Q. He X. Yu J. Feng Y. (2019). Extracellular DNA enhances the formation and stability of symplasmata in *Pantoea agglomerans* YS19. J. Gen. Appl. Microbiol. 65, 11–17. 10.2323/jgam.2018.03.002 30185735

