# Supplementary material for: Whole genome sequencing and characterization of Pantoea agglomerans DBM 3797, endophyte, isolated from fresh hop (Humulus lupulus L.)
Source: Front Microbiol. 2024 Feb 8;15:1305338. doi: 10.3389/fmicb.2024.1305338 (PMC10882544; doi:10.3389/fmicb.2024.1305338)
Supplement: Supplementary file 1 [file Data_Sheet_1.docx]

Supplementary Material

**Supplementary Table 1 Summary of clusters of orthologous groups (COG) of *P. agglomerans* DBM 3797**

|  |  | **Chromosome** | | **Plasmid pPA_DBM3797_1** | | **Plasmid pPA_DBM3797_2** | |
| --- | --- | --- | --- | --- | --- | --- | --- |
| **COG category** | **Description** | **Gene count [-]** | **Relative abundance [%]** | **Gene count [-]** | **Relative abundance [%]** | **Gene count [-]** | **Relative abundance [%]** |
| J | Translation, ribosomal structure and biogenesis | 264 | 7.14 | 13 | 2.46 | 2 | 1.34 |
| A | RNA processing and modification | 1 | 0.03 | 0 | 0.00 | 0 | 0.00 |
| K | Transcription | 261 | 7.06 | 78 | 14.74 | 15 | 10.07 |
| L | Replication, recombination and repair | 138 | 3.73 | 8 | 1.51 | 3 | 2.01 |
| B | Chromatin structure and dynamics | 0 | 0.00 | 0 | 0.00 | 0 | 0.00 |
| D | Energy production and conversion | 52 | 1.41 | 2 | 0.38 | 2 | 1.34 |
| Y | Nuclear structure | 0 | 0.00 | 0 | 0.00 | 0 | 0.00 |
| V | Defense mechanisms | 81 | 2.19 | 11 | 2.08 | 15 | 10.07 |
| T | Signal transduction mechanisms | 159 | 4.30 | 20 | 3.78 | 20 | 13.42 |
| M | Cell wall/membrane/envelope biogenesis | 268 | 7.25 | 17 | 3.21 | 11 | 7.38 |
| N | Cell motility | 70 | 1.89 | 5 | 0.95 | 4 | 2.68 |
| Z | Cytoskeleton | 0 | 0.00 | 0 | 0.00 | 0 | 0.00 |
| W | Extracellular structures | 10 | 0.27 | 2 | 0.38 | 1 | 0.67 |
| U | Intracellular trafficking, secretion, and vesicular transport | 77 | 2.08 | 3 | 0.57 | 0 | 0.00 |
| O | Post-translational modification, protein turnover, and chaperones | 123 | 3.33 | 12 | 2.27 | 3 | 2.01 |
| X | Mobilome: prophages, transposons | 33 | 0.89 | 2 | 0.38 | 1 | 0.67 |
| C | Energy production and conversion | 167 | 4.51 | 22 | 4.16 | 2 | 1.34 |
| G | Carbohydrate transport and metabolism | 321 | 8.68 | 69 | 13.04 | 14 | 9.40 |
| E | Amino acid transport and metabolism | 323 | 8.73 | 37 | 6.99 | 2 | 1.34 |
| F | Nucleotide transport and metabolism | 101 | 2.73 | 8 | 1.51 | 0 | 0.00 |
| H | Coenzyme transport and metabolism | 165 | 4.46 | 31 | 5.86 | 4 | 2.68 |
| I | Lipid transport and metabolism | 125 | 3.38 | 13 | 2.46 | 3 | 2.01 |
| P | Inorganic ion transport and metabolism | 184 | 4.97 | 32 | 6.05 | 3 | 2.01 |
| Q | Secondary metabolites biosynthesis, transport, and catabolism | 39 | 1.05 | 11 | 2.08 | 3 | 2.01 |
| R | General function prediction only | 164 | 4.43 | 41 | 7.75 | 14 | 9.40 |
| S | Function unknown | 154 | 4.16 | 26 | 4.91 | 8 | 5.37 |
| unknown |  | 419 | 11.33 | 66 | 12.48 | 19 | 12.75 |

**Supplementary Table 2 Restriction-modification (R-M) systems of *P. agglomerans* DBM 3797**

| **Gene locus** | **Gene** | **Most similar** | **Specificity** | **Name** |
| --- | --- | --- | --- | --- |
| **Type I** | | | | |
| LKW31_16825 |  | N-6 DNA methylase |  |  |
| LKW31_16830 | R | EmaJC163ORFBP (98% identity) |  | Pag3797ORF16840P |
| LKW31_16835 | S | S.ScoYN2ORF16795P (95% identity) |  | S.Pag3797ORF16840P |
| LKW31_16840 | M | M2.Yen3502ORF590P (95% identity) |  | M2.Pag3797ORF16840P |
| LKW31_16845 | M | M1.EcosA33III (98% identity) |  | M1.Pag3797ORF16840P |
| LKW31_16850 |  | helix-turn-helix transcriptional regulator |  |  |
| **Type II** | | | | |
| LKW31_1865 |  | SPOR domain-containing protein |  |  |
| LKW31_1870 | M | M.PagUAEU18DamP (100% identity) | GATC | M.Pag3797DamP |
| LKW31_1875 |  | ribulose-phosphate 3-epimerase |  |  |
| LKW31_3125 |  | TraR/DksA family transcriptional regulator |  |  |
| LKW31_3130 | M | M.Pag4188ORF7270P (94% identity) |  | M.Pag3797ORF3130P |
| LKW31_3135 |  | replication endonuclease |  |  |
| LKW31_7880 |  | Arc family DNA-binding protein |  |  |
| LKW31_7885 | M | M.PagIG1DcmP (99% identity) | CCWGG | M.Pag3797DcmP |
| LKW31_7890 | V | V.PanGB1DcmP (100% identity) |  | V.Pag3797DcmP |
| LKW31_7895 |  | 91 aa hypothetical protein |  |  |

**Supplementary Table 3 Antibiotic resistance genes of *P. agglomerans* DBM 3797**

| **Locus** | **ARO Term** | **Detection Criteria** | **AMR Gene Family** | **Drug Class** | **Resistance Mechanism** | **% Identity of Matching Region** | **% Length of Reference Sequence** |
| --- | --- | --- | --- | --- | --- | --- | --- |
| **Chromosome** | | | | | | | |
| LKW31_01945 | CRP | protein homolog model | resistance-nodulation-cell division (RND) antibiotic efflux pump | macrolide antibiotic, fluoroquinolone antibiotic, penam | antibiotic efflux | 98.57 | 100.00 |
| LKW31_04605 | rsmA | protein homolog model | resistance-nodulation-cell division (RND) antibiotic efflux pump | fluoroquinolone antibiotic, diaminopyrimidine antibiotic, phenicol antibiotic | antibiotic efflux | 89.66 | 100.00 |
| LKW31_04800 | *Klebsiella pneumoniae* KpnH | protein homolog model | major facilitator superfamily (MFS) antibiotic efflux pump | macrolide antibiotic, fluoroquinolone antibiotic, aminoglycoside antibiotic, carbapenem, cephalosporin, penam, peptide antibiotic, penem | antibiotic efflux | 88.21 | 99.80 |
| LKW31_07665 | FosA8 | protein homolog model | fosfomycin thiol transferase | phosphonic acid antibiotic | antibiotic inactivation | 55.88 | 93.62 |
| LKW31_08575 | *Klebsiella pneumoniae* KpnF | protein homolog model | small multidrug resistance (SMR) antibiotic efflux pump | macrolide antibiotic, aminoglycoside antibiotic, cephalosporin, tetracycline antibiotic, peptide antibiotic, rifamycin antibiotic, disinfecting agents and antiseptics | antibiotic efflux | 75.73 | 100.00 |
| LKW31_10340 | adeF | protein homolog model | resistance-nodulation-cell division (RND) antibiotic efflux pump | fluoroquinolone antibiotic, tetracycline antibiotic | antibiotic efflux | 60.8 | 99.15 |
| LKW31_14650 | adeF | protein homolog model | resistance-nodulation-cell division (RND) antibiotic efflux pump | fluoroquinolone antibiotic, tetracycline antibiotic | antibiotic efflux | 42.06 | 99.24 |
| LKW31_00020 | *Morganella morganii* gyrB conferring resistance to fluoroquinolones | protein variant model | fluoroquinolone resistant gyrB | fluoroquinolone antibiotic | antibiotic target alteration | 80.72 | 99.75 |
| LKW31_02070 | *Escherichia coli* EF-Tu mutants conferring resistance to Pulvomycin | protein variant model | elfamycin resistant EF-Tu | elfamycin antibiotic | antibiotic target alteration | 91.09 | 96.33 |
| LKW31_16160 | *Haemophilus influenzae* PBP3 conferring resistance to beta-lactam antibiotics | protein variant model | Penicillin-binding protein mutations conferring resistance to beta-lactam antibiotics | cephalosporin, cephamycin, penam | antibiotic target alteration | 53.19 | 96.39 |
| LKW31_18275 | *Escherichia coli* EF-Tu mutants conferring resistance to Pulvomycin | protein variant model | elfamycin resistant EF-Tu | elfamycin antibiotic | antibiotic target alteration | 89.82 | 96.33 |
| **Plasmid**  pPA_DBM3797_2 | | | | | | | |
| LKW31_22010 | ArnT | protein homolog model | pmr phosphoethanolamine transferase | peptide antibiotic | antibiotic target alteration | 60.41 | 100.73 |

**Supplementary Table 4 – The complete list of strains used for *P. agglomerans* phylogenetic tree (Figure 2)**

1 GCF_019048385.1 Pantoea agglomerans FDAARGOS 1447

2 GCF_943184895.1 Pantoea agglomerans DAPP-PG734

3 GCF_031888365.1 Pantoea agglomerans SUH1

4 GCF_031887785.1 Pantoea agglomerans T88c

5 GCF_031890185.1 Pantoea agglomerans BH6c

6 GCF_031891025.1 Pantoea agglomerans AJ2b

7 GCF_001709315.1 Pantoea agglomerans C410P1

8 GCF_017474165.1 Pantoea agglomerans AR1a

9 GCF_021559955.1 Pantoea agglomerans ZJU23

10 GCF_029016125.1 Pantoea agglomerans CB1

11 GCF_003704305.1 Pantoea agglomerans TH81

12 GCF_031890505.1 Pantoea agglomerans AR5

13 GCF_022966105.1 Pantoea agglomerans Pa58

14 GCF_031890525.1 Pantoea agglomerans AR24

15 GCF_020783495.1 Pantoea agglomerans DBM 3797

16 GCF_031889005.1 Pantoea agglomerans ROTS050421

17 GCF_004117135.1 Pantoea agglomerans CFSAN047154

18 GCF_004136415.1 Pantoea agglomerans CFSAN047153

19 GCF_026625085.1 Pantoea agglomerans AB378

20 GCF_031890205.1 Pantoea agglomerans AR8b

21 GCF_031889865.1 Pantoea agglomerans FC61912-B

22 GCF_003860325.1 Pantoea agglomerans L15

23 GCF_009762725.1 Pantoea agglomerans ASB05

24 GCF_024799805.1 Pantoea agglomerans CHTF15

25 GCF_030710645.1 Pantoea agglomerans NBBC-01

26 GCF_031889605.1 Pantoea agglomerans MMD61212-C

27 GCF_001662025.2 Pantoea agglomerans 4188

28 GCF_001661985.2 Pantoea agglomerans 824-1

29 GCF_021609925.1 Pantoea agglomerans PSV1-7

30 GCF_010523255.1 Pantoea agglomerans UAEU18

31 GCF_023676805.1 Pantoea agglomerans CPHN 2

32 GCF_032200625.1 Pantoea agglomerans 1.2.4

33 GCF_001598475.1 Pantoea agglomerans NBRC 102470

34 GCF_900454505.1 Pantoea agglomerans NCTC10500

35 GCF_030815835.1 Pantoea agglomerans W2I1

36 GCF_030814785.1 Pantoea agglomerans PvP026

37 GCF_001597625.1 Pantoea agglomerans 3

38 GCF_009765475.1 Pantoea agglomerans BAV 2934

39 GCF_030177195.1 Pantoea agglomerans 9Rz4

40 GCF_000731125.1 Pantoea agglomerans 190

41 GCF_900182855.1 Pantoea agglomerans GR13

42 GCF_000743785.2 Pantoea agglomerans 4

43 GCF_900454395.1 Pantoea agglomerans NCTC10601

44 GCF_014354155.1 Pantoea agglomerans Pa21-13

45 GCF_009759885.1 Pantoea agglomerans C1

46 GCF_014839425.1 Pantoea agglomerans CFBP13616

47 GCF_014354165.1 Pantoea agglomerans Pa17-5

48 GCF_014138175.1 Pantoea agglomerans 62e

49 GCF_014138195.1 Pantoea agglomerans 62d

50 GCF_010667925.1 Pantoea agglomerans T1

51 GCF_010667775.1 Pantoea agglomerans T6

52 GCF_023614195.1 Pantoea agglomerans CCNPK 873

53 GCF_014839525.1 Pantoea agglomerans CFBP13593

54 GCF_019969965.1 Pantoea agglomerans OVA06A

55 GCF_010667975.1 Pantoea agglomerans B3

56 GCF_010667995.1 Pantoea agglomerans R1

57 GCF_014842695.1 Pantoea agglomerans CFBP13600

58 GCF_000627115.1 Pantoea agglomerans RIT273

59 GCF_014353795.1 Pantoea agglomerans Pa39-7

60 GCF_014839545.1 Pantoea agglomerans CFBP13583

61 GCF_014838815.1 Pantoea agglomerans CFBP8800

62 GCF_014839125.1 Pantoea agglomerans CFBP8784

63 GCF_010667865.1 Pantoea agglomerans T5

64 GCF_010667845.1 Pantoea agglomerans T2

65 GCF_010667855.1 Pantoea agglomerans T3

66 GCF_010667895.1 Pantoea agglomerans T4

67 GCF_010667945.1 Pantoea agglomerans R5

68 GCF_000757415.2 Pantoea agglomerans MP2

69 GCF_010667965.1 Pantoea agglomerans A2

70 GCF_002222515.1 Pantoea agglomerans JM1

71 GCF_014838975.1 Pantoea agglomerans CFBP8792

72 GCF_900185875.1 Pantoea agglomerans DSM 3493

73 GCF_014138245.1 Pantoea agglomerans MR10

74 GCF_010667985.1 Pantoea agglomerans B1

75 GCF_030815435.1 Pantoea agglomerans DSM 3493_2

76 GCF_014839115.1 Pantoea agglomerans CFBP13569

77 GCF_014138205.1 Pantoea agglomerans MR19

78 GCF_003258435.1 Pantoea agglomerans Bl3

79 GCF_004793995.1 Pantoea agglomerans Bl3

80 GCF_014354005.1 Pantoea agglomerans E325-ad2

81 GCF_001288285.1 Pantoea agglomerans P10c

82 GCF_009668105.1 Pantoea agglomerans RIT710

83 GCF_905332865.1 Pantoea agglomerans RSO7

84 GCF_014839175.1 Pantoea agglomerans CFBP8783

85 GCF_000952095.1 Pantoea agglomerans GB1

86 GCF_014353755.1 Pantoea agglomerans Pa39-5

87 GCF_025642955.1 Pantoea agglomerans RIT-PI-T

88 GCF_029838845.1 Pantoea agglomerans GD03966

89 GCF_014353845.1 Pantoea agglomerans E325-699

90 GCF_007050955.1 Pantoea agglomerans K1

91 GCF_012241415.1 Pantoea agglomerans KM1

92 GCF_015711555.1 Pantoea agglomerans NC

93 GCF_014353815.1 Pantoea agglomerans Pa39-3

94 GCF_014354135.1 Pantoea agglomerans Pa21-3

95 GCF_014841465.1 Pantoea agglomerans CFBP8774

96 GCF_014839315.1 Pantoea agglomerans CFBP13731

97 GCF_014353705.1 Pantoea agglomerans Pa31-3

98 GCF_014354145.1 Pantoea agglomerans Pa21-5

99 GCF_014839205.1 Pantoea agglomerans CFBP8756

100 GCF_017348995.1 Pantoea agglomerans MM2021_7

101 GCF_013403235.1 Pantoea agglomerans FJII-L5-SW-P2

102 GCF_029623495.1 Pantoea agglomerans RIT-To-3

103 GCF_000220605.1 Pantoea agglomerans Sl1_M5

104 GCF_014353945.1 Pantoea agglomerans Pa39-23

105 GCF_014353935.1 Pantoea agglomerans E325-ad1

106 GCF_014354045.1 Pantoea agglomerans Pa31-4

107 GCF_014839325.1 Pantoea agglomerans CFBP13709

108 GCF_014353955.1 Pantoea agglomerans E325-754

109 GCF_014354035.1 Pantoea agglomerans Pa39-21

110 GCF_014353865.1 Pantoea agglomerans E325

111 GCF_004344785.1 Pantoea agglomerans ES418

112 GCF_022585235.1 Pantoea agglomerans 20TX0122

113 GCF_014839015.1 Pantoea agglomerans CFBP8786

114 GCF_014354055.1 Pantoea agglomerans Pa39-14

115 GCF_023508185.1 Pantoea agglomerans ICMP 1087

116 GCF_014353835.1 Pantoea agglomerans E325-705

117 GCF_014353995.1 Pantoea agglomerans E325-750

118 GCF_014354235.1 Pantoea agglomerans Pa17-1

119 GCF_014353745.1 Pantoea agglomerans Pa39-1

120 GCF_030433795.1 Pantoea agglomerans Pan8

121 GCF_005233495.1 Pantoea agglomerans CFBP13505

122 GCF_014839265.1 Pantoea agglomerans CFBP13566

123 GCF_005233795.1 Pantoea agglomerans CFBP13516

124 GCF_014839085.1 Pantoea agglomerans CFBP8785

125 GCF_014838905.1 Pantoea agglomerans CFBP8791

126 GCF_005233805.1 Pantoea agglomerans CFBP13532

127 GCF_014354065.1 Pantoea agglomerans Pa21-15

128 GCF_017315165.1 Pantoea agglomerans ANP8

129 GCF_014353895.1 Pantoea agglomerans Pa39-27

130 GCF_000814075.1 Pantoea agglomerans LMAE-2

131 GCF_013201565.1 Pantoea agglomerans DOAB1048

132 GCF_003369485.1 Pantoea agglomerans BD 1212

133 GCF_002157425.2 Pantoea agglomerans P5

134 GCF_000710215.1 Pantoea agglomerans DAPP-PG734

135 GCF_003369505.1 Pantoea agglomerans BD 1274

136 GCF_015025115.1 Pantoea agglomerans R6

137 GCF_000475055.1 Pantoea agglomerans Tx10

**Supplementary Table 5 Candidate genes for plant growth promoting activity**

*While most of the genes are located on a chromosome, only plasmid pPA_DBM3797_1 location was indicated.

| **Gene locus** | **Gene product annotation** | **Gene abbreviation** | **Location^*^** |
| --- | --- | --- | --- |
| **Amylase production** | | |  |
| LKW31_01760 | alpha-amylase family glycosyl hydrolase |  |  |
|  |  |  |  |
| LKW31_02980 | glucoamylase family protein |  |  |
|  |  |  |  |
| LKW31_08125 | alpha-amylase |  |  |
|  |  |  |  |
| **Pectinase** | | |  |
| LKW31_11050 | 5-dehydro-4-deoxy-D-glucuronate isomerase |  |  |
|  |  |  |  |
| **Cellulase** | | | |
| LKW31_19555 | PTS cellobiose/arbutin/salicin transporter subunit IIBC |  | *Plasmid* |
|  |  |  |  |
| **siderophore related** | | |  |
| LKW31_03785 | siderophore-interacting protein |  |  |
|  |  |  |  |
| LKW31_11745 | TonB-dependent siderophore receptor |  |  |
|  |  |  |  |
| LKW31_16615 | siderophore-iron reductase FhuF | *fhuF* |  |
|  |  |  |  |
| LKW31_17240 | TonB-dependent siderophore receptor |  |  |
|  |  |  |  |
| LKW31_17255 | enterobactin synthase subunit F |  |  |
| LKW31_17260 | ATP-binding cassette domain-containing protein |  |  |
| LKW31_17265 | iron-enterobactin ABC transporter permease |  |  |
| LKW31_17270 | Fe(3+)-siderophore ABC transporter permease |  |  |
| LKW31_17275 | enterobactin transporter EntS |  |  |
| LKW31_17280 | Fe2+-enterobactin ABC transporter substrate-binding protein |  |  |
|  |  |  |  |
| LKW31_19325 | TonB-dependent siderophore receptor |  | *Plasmid* |
|  |  |  |  |
| LKW31_19865 | TonB-dependent siderophore receptor" |  | *Plasmid* |
|  |  |  |  |
| LKW31_19880 | ferric-rhodotorulic acid/ferric-coprogen receptor FhuE | *fhuE* | *Plasmid* |
| **indole-acetic acid related** | | |  |
| LKW31_05775 | indolepyruvate decarboxylase | *ipdC* |  |
|  |  |  |  |
| **Motility, symplasmata formation, biofilm formation** | | |  |
| **exopolysaccharide formation** | | |  |
| LKW31_01775 | exopolysaccharide production protein YjbE | *yjbE* |  |
|  |  |  |  |
| LKW31_18100 | exopolysaccharide production protein YjbE | *yjbE* |  |
| LKW31_18105 | YjbF family lipoprotein | *yjbF* |  |
| LKW31_18110 | capsule biosynthesis GfcC family protein |  |  |
| LKW31_18115 | YjbH domain-containing protein | *yjbH* |  |
|  |  |  |  |
| **Flagella formation and motility** | | |  |
| LKW31_06150 | flagella biosynthesis regulator Flk | *flK* |  |
|  |  |  |  |
| LKW31_06305 | transcriptional regulator LrhA | *lrhA* |  |
|  |  |  |  |
| LKW31_07945 | flagellar biosynthesis protein FliR | *fliR* |  |
| LKW31_07950 | flagellar biosynthesis protein FliQ | *fliQ* |  |
| LKW31_07955 | flagellar type III secretion system pore protein FliP | *fliP* |  |
| LKW31_07960 | flagellar biosynthetic protein FliO | *fliO* |  |
| LKW31_07965 | flagellar motor switch protein FliN | *fliN* |  |
| LKW31_07970 | flagellar motor switch protein FliM | *fliM* |  |
| LKW31_07975 | flagellar basal body-associated protein FliL | *fliL* |  |
| LKW31_07980 | flagellar hook-length control protein FliK | *fliK* |  |
| LKW31_07985 | flagellar export protein FliJ | *fliJ* |  |
| LKW31_07990 | flagellar protein export ATPase FliI | *fliI* |  |
| LKW31_07995 | flagellar assembly protein FliH | *fliH* |  |
| LKW31_08000 | flagellar motor switch protein FliG | *fliG* |  |
| LKW31_08005 | flagellar basal-body MS-ring/collar protein FliF | *fliF* |  |
| LKW31_08010 | flagellar hook-basal body complex protein FliE | *fliE* |  |
|  |  |  |  |
| LKW31_08130 | flagella biosynthesis regulatory protein FliT | *fliT* |  |
| LKW31_08135 | flagellar export chaperone FliS | *fliS* |  |
| LKW31_08140 | flagellar filament capping protein FliD | *fliD* |  |
| LKW31_08145 | FliC/FljB family flagellin | *fliC/fljB* |  |
| LKW31_08150 | flagellin lysine-N-methylase |  |  |
|  |  |  |  |
| LKW31_08160 | RNA polymerase sigma factor FliA | *fliA* |  |
| LKW31_08165 | flagella biosynthesis regulatory protein FliZ | *fliZ* |  |
|  |  |  |  |
| LKW31_08265 | flagellar transcriptional regulator FlhD | *flhD* |  |
| LKW31_08270 | flagellar transcriptional regulator FlhC | *flhC* |  |
| LKW31_08275 | flagellar motor stator protein MotA | *motA* |  |
| LKW31_08280 | flagellar motor protein MotB | *motB* |  |
| LKW31_08285 | chemotaxis protein CheA | *cheA* |  |
| LKW31_08290 | chemotaxis protein CheW | *cheW* |  |
| LKW31_08295 | methyl-accepting chemotaxis protein |  |  |
| LKW31_08300 | methyl-accepting chemotaxis protein |  |  |
| LKW31_08305 | methyl-accepting chemotaxis protein |  |  |
| LKW31_08310 | protein-glutamate O-methyltransferase CheR | *cheR* |  |
| LKW31_08315 | chemotaxis response regulator protein-glutamate methylesterase |  |  |
| LKW31_08320 | chemotaxis response regulator CheY | *cheY* |  |
| LKW31_08325 | protein phosphatase CheZ | *cheZ* |  |
| LKW31_08330 | flagellar biosynthesis protein FlhB | *flhB* |  |
| LKW31_08335 | flagellar biosynthesis protein FlhA | *flhA* |  |
| LKW31_08340 | flagellar protein FlhE | *flhE* |  |
|  |  |  |  |
| **Quorum sensing** | | |  |
| LKW31_16760 | LuxR family transcriptional regulator | *luxR* |  |
| LKW31_16765 | acyl-homoserine-lactone synthase |  |  |
|  |  |  |  |
| LKW31_18480 | homoserine/homoserine lactone efflux protein |  |  |
|  |  |  |  |
| LKW31_12510 | acyl-homoserine-lactone synthase |  |  |
|  |  |  |  |
| **Phosphate solubilization related** | | |  |
| **Phosphonate metabolism** | | |  |
| LKW31_07670 | phosphonate metabolism transcriptional regulator | *phnF* |  |
| LKW31_07675 | phosphonate C-P lyase system protein | *phnG* |  |
| LKW31_07680 | phosphonate C-P lyase system protein | *phnH* |  |
| LKW31_07685 | carbon-phosphorus lyase complex subunit | *phnI* |  |
| LKW31_07690 | alpha-D-ribose 1-methylphosphonate 5-phosphate C-P-lyase | *phnJ* |  |
| LKW31_07695 | phosphonate C-P lyase system protein | *phnK* |  |
| LKW31_07700 | phosphonate C-P lyase system protein | *phnL* |  |
| LKW31_07705 | alpha-D-ribose 1-methylphosphonate 5-triphosphate diphosphatase | *phnM* |  |
| LKW31_07710 | ribose 1,5-bisphosphokinase | *phnN* |  |
| LKW31_07715 | phosphonate metabolism protein | *phnP* |  |
| LKW31_07720 | phosphonate ABC transporter ATP-binding protein | *phnC* |  |
| LKW31_07725 | phosphonate ABC transporter substrate-binding protein | *phnD* |  |
| LKW31_07730 | phosphonate ABC transporter, permease protein | *phnE* |  |
| LKW31_07735 | phosphonate ABC transporter, permease protein | *phnE* |  |
|  |  |  |  |
| ***Phosphate transporters pst*** | | |  |
| LKW31_00190 | phosphate signaling complex protein | *phoU* |  |
| LKW31_00195 | phosphate ABC transporter ATP-binding protein | *pstB* |  |
| LKW31_00200 | phosphate ABC transporter permease PstA | *pstA* |  |
| LKW31_00205 | phosphate ABC transporter permease PstC | *pstC* |  |
| LKW31_00210 | phosphate ABC transporter substrate-binding protein PstS | *pstS* |  |
|  |  |  |  |
| LKW31_15025 | PstS family phosphate ABC transporter substrate-binding protein |  |  |
| LKW31_15030 | phosphate regulon sensor histidine kinase PhoR | *phoR* |  |
| LKW31_15035 | phosphate response regulator transcription factor | *phoB* |  |
|  |  |  |  |
| **Inorganic phosphate processing** | | | |
| LKW31_01325 | inorganic phosphate transporter PitA | *pitA* |  |
|  |  |  |  |
| LKW31_02710 | inorganic diphosphatase |  |  |
|  |  |  |  |
| LKW31_03395 | inorganic triphosphatase |  |  |
|  |  |  |  |
| LKW31_13535 | inorganic phosphate transporter |  |  |
|  |  |  |  |
| **ACC deaminase** | | |  |
| LKW31_17015 | 2-iminobutanoate/2-iminopropanoate deaminase | *ridA* |  |
|  |  |  |  |
| **Carotenoid synthesis** | | | |
| LKW31_20840 | lycopene beta-cyclase CrtY | *crtY* | *Plasmid* |
| LKW31_20845 | phytoene desaturase |  | *Plasmid* |
| LKW31_20850 | phytoene/squalene synthase family protein |  | *Plasmid* |
| LKW31_20855 | sterol desaturase family protein |  | *Plasmid* |
|  |  |  |  |
|  |  |  |  |

**Supplementary Figure 1**
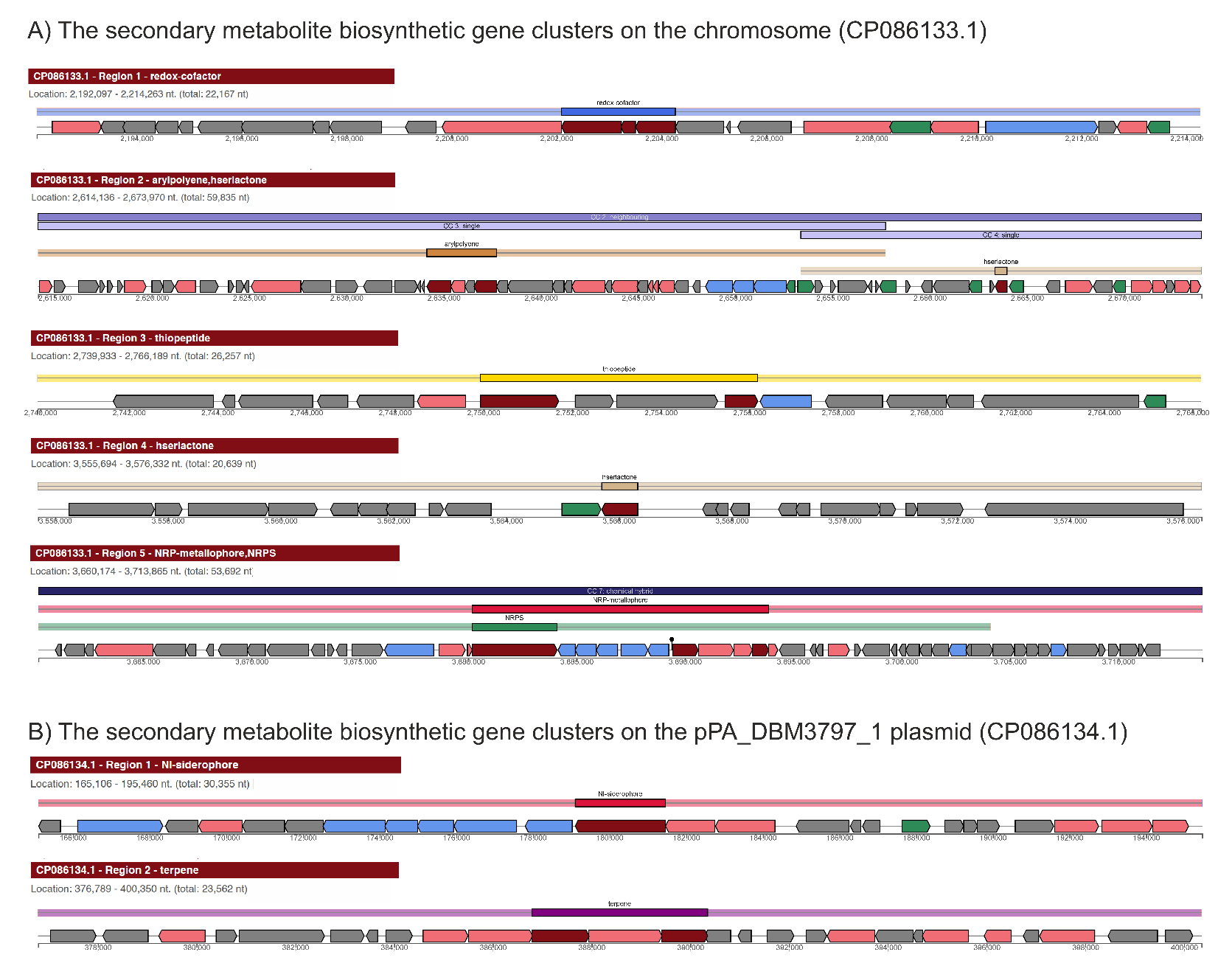
 **Biosynthetic clusters on (A) the chromosome and (B) pPA_DBM3797_1 plasmid**


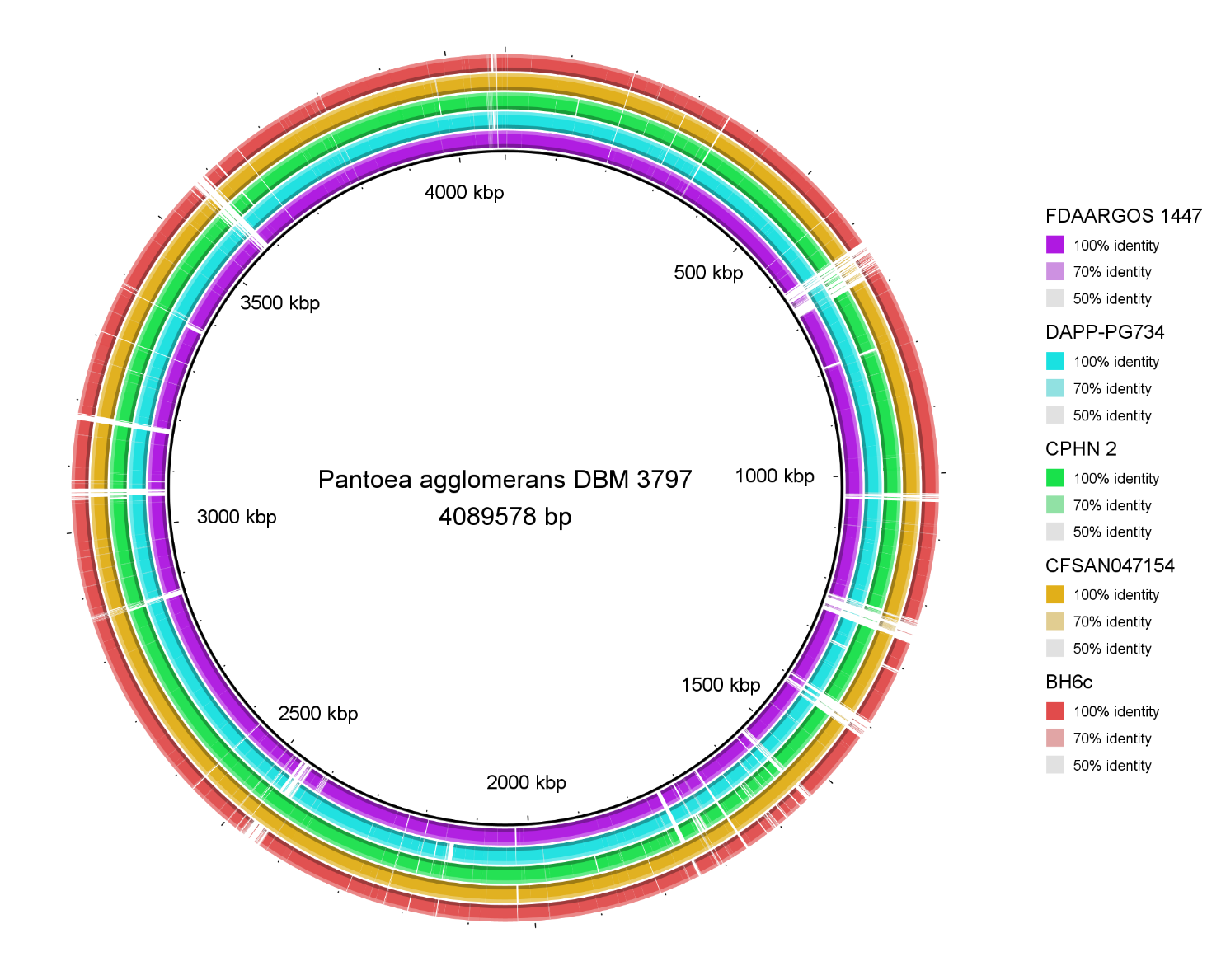


**Supplementary Figure 2 Comparison of chromosomal sequences of *P. agglomerans* DBM 3797 with selected pathogenic (FDAARGOS 1447 and BH6c) and non-pathogenic (DAPP-PG734, CPHN 2, CFSAN047154) *P. agglomerans* strains.**


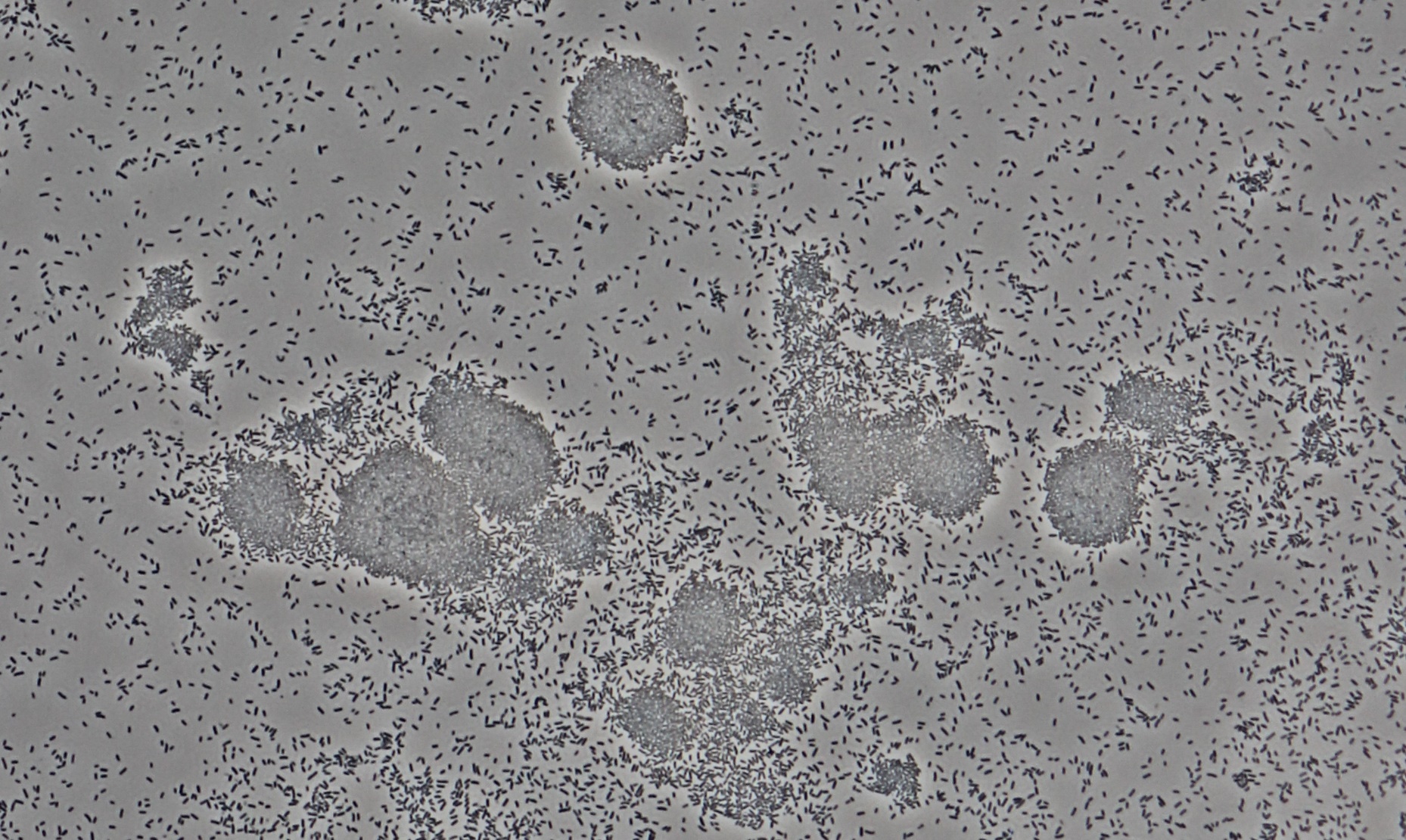


**Supplementary Figure 3 Symplasmata formation after growth of *P. agglomerans* DBM 3797 in LB medium**

Growth conditions: 30°C, rotary shaker (150 rpm), Erlenmeyer flasks, 24h


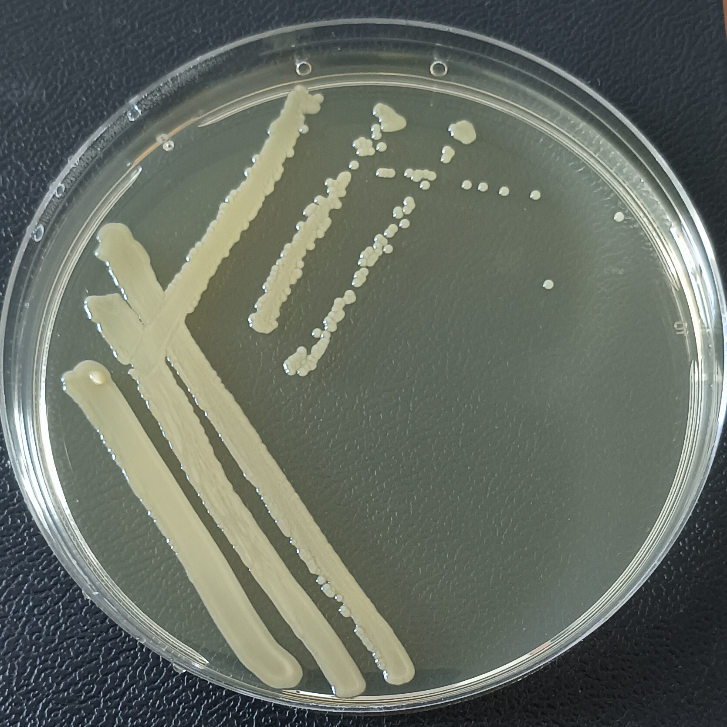


**Supplementary Figure 4 Colonies of *P. agglomerans* DBM 3797 grown on LB agar after 48 h incubation at 30°C.**

**Supplementary** **Figure 5 Comparison of aerobic and anaerobic growth in PGM medium containing 20 g/l of glucose**
